# Machine Learning‐Based Prediction of Poor Outcomes in Intracerebral Hemorrhage: A Systematic Review and Meta‐Analysis

**DOI:** 10.1002/brb3.71572

**Published:** 2026-07-31

**Authors:** Qi Deng, Weitao Cheng, Hui Lu, Nan Fei, Rong He, Yuanyuan Chen, Jianlong Bi, Wenluo Zhang

**Affiliations:** ^1^ Neurology Department PKUCare Rehabilitation Hospital Beijing China; ^2^ Department of Neurosurgery Xuanwu Hospital Capital Medical University Beijing China; ^3^ Neurology Department Aerospace Central Hospital Beijing China; ^4^ Rehab Department PKUCare Rehabilitation Hospital Beijing China; ^5^ Intensive Care Unit PKUCare Rehabilitation Hospital Beijing China

**Keywords:** intracerebral hemorrhage, machine learning, prognosis, systematic review, meta‐analysis, hematoma expansion, functional outcome

## Abstract

**Background:**

Spontaneous intracerebral hemorrhage (ICH) is associated with high risks of mortality and disability, yet early and accurate outcome prediction remains challenging. This study systematically evaluated the performance of machine learning (ML) models in predicting key adverse outcomes (hematoma expansion [HE], poor functional outcome, mortality) in ICH, aiming to provide consolidated evidence for future research and clinical translation.

**Methods:**

We systematically searched PubMed, Embase, Web of Science, and Cochrane Library up to September 2025. Studies developing and validating ML models for predicting HE, poor functional outcome (modified Rankin Scale 3–6), or mortality in adults with spontaneous ICH were included. Pooled concordance index (C‐index), sensitivity, and specificity were calculated using a random‐effects or bivariate model.

**Results:**

Eighty‐three studies (involving at least 136,840 patients) were included. Meta‐analysis of model performance, derived predominantly from internal validation set, demonstrated that models integrating both clinical and radiomics features achieved the highest discriminative performance across key prognostic prediction tasks: predicting HE (pooled C‐index 0.822, 95% confidence interval [CI] 0.789–0.855), poor functional outcome (C‐index 0.850, 95% CI 0.830–0.869), and mortality (C‐index 0.860, 95% CI 0.809–0.911). These findings should be interpreted with caution, as true external validation remains sparse. Logistic regression exhibited performance comparable to more complex ML algorithms.

**Conclusions:**

ML models, particularly integrated clinical‐radiomics models, demonstrate strong performance for the prediction of outcomes in ICH. They hold significant potential to enhance risk stratification and guide personalized management, pending further validation in diverse cohorts.

## Graphical abstract

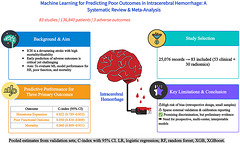

**Abbreviations**
AUC: area under the curve
CI: confidence interval
CT: computed tomography
DL: deep learning
GCS
: Glasgow Coma Scale
GOS: Glasgow Outcome Scale
HE: hematoma expansion
HT: hemorrhagic transformation
ICH: intracerebral hemorrhage
LASSO: Least Absolute Shrinkage and Selection Operator
LR: logistic regression
ML: machine learning
MRI: magnetic resonance imaging
mRS: modified Rankin Scale
NCCT: non‐contrast computed tomography
RF: Random Forest
RICH: recurrent intracerebral hemorrhage
SHAP: SHapley Additive exPlanations
SVM: Support Vector Machine
XAI: explainable artificial intelligence
XGB: XGBoost

## 1 Introduction

Intracerebral hemorrhage (ICH) is a devastating form of stroke characterized by bleeding into the brain parenchyma (He et al. 2024). It is a devastating cerebrovascular event with high mortality and disability rates, posing a significant global public health challenge, particularly to the middle‐aged and elderly populations. According to the systematic analysis for the Global Burden of Disease Study 2024, stroke remains a leading cause of death and disability worldwide, with ICH accounting for a substantial portion of this burden (He et al. 2024). Contemporary epidemiological studies have revealed notable geographical disparities. For instance, in the United States, the nationwide incidence of primary ICH increased by 11% from 2004 to 2018, with a particularly marked rise observed among young and middle‐aged adults (18–64 years), indicating a trend toward younger onset (Bako et al. 2022). This trend is juxtaposed with data from European studies, which project a continued increase in the absolute number of ICH‐related deaths by 2050, largely driven by population aging (Wafa et al. 2024). Despite advancements in acute care management, the long‐term prognosis for ICH survivors remains poor. A comprehensive study on long‐term survival after ICH demonstrated that mortality rates remain substantially elevated compared to the general population, with a 5‐year survival rate of approximately 65%; however, this figure masks a significant risk, as the cumulative mortality can be as high as 35%, representing a 13‐fold increased risk of death compared to matched controls, primarily due to vascular and nonvascular causes (Carlsson et al. 2021). Therefore, ICH continues to be a major disease burden that severely threatens the health and lives of adults globally, underscoring the critical need for improved prognostic prediction strategies.

Current management of ICH primarily focuses on rapid hemorrhage control, blood pressure management, and supportive care to mitigate secondary brain injury (Q. Li et al. 2024). Guidelines recommend intensive blood pressure lowering and, in select cases, surgical interventions such as minimally invasive surgery or endoscopic evacuation to improve outcomes (Mansoor et al. 2024). Despite these interventions, a substantial proportion of patients experience a poor prognosis. Hematoma expansion (HE), a key determinant of early deterioration, occurs in approximately 30% of patients and is strongly associated with worse functional outcomes (Morotti et al. 2023). Furthermore, studies indicate that nearly 60% of survivors face severe disability (modified Rankin Scale [mRS] score 4–5) at 90 days, and long‐term mortality remains significantly elevated (Carlsson et al. 2021; Sprügel et al. 2021). Consequently, international guidelines emphasize the importance of early and intensive rehabilitative strategies to maximize functional recovery (Campbell and Khatri 2020). However, effective tools for the precise early prediction and preemptive management of these specific adverse outcomes are still lacking, highlighting a critical gap in personalized patient care.

In recent years, machine learning (ML) has garnered significant attention in clinical research for its ability to identify complex, nonlinear patterns from high‐dimensional data, thereby facilitating the discovery of novel biomarkers for targeted intervention and refined prognostic stratification (Guo et al. 2022). Within the field of ICH, ML applications have demonstrated considerable promise. For instance, deep learning (DL) survival models, such as fICHNet, have shown superior accuracy over traditional clinical scores in predicting long‐term functional outcomes from initial non‐contrast computed tomography (NCCT) scans (Y. Chen, Rivier, et al. 2025). Similarly, ML‐based radiomics models have been developed to accurately predict the risk of HE, a critical early determinant of poor prognosis (Haider et al. 2023). Furthermore, ensemble models integrating clinical and imaging data have been constructed to forecast in‐hospital mortality with high discrimination (S. Y. Peng et al. 2010). Extensive research has been conducted in the field utilizing a diversity of modeling approaches, feature extraction techniques, and outcome definitions. However, the absence of a comprehensive, evidence‐based synthesis makes it challenging to ascertain the overall predictive value of ML and to guide the development or updating of robust risk assessment tools. Therefore, we conducted this systematic review and meta‐analysis to critically evaluate the performance of ML‐based models for predicting adverse outcomes, specifically poor functional outcome, HE, and mortality, in patients with ICH, aiming to provide consolidated evidence for future research and clinical translation.

## 2 Methods

### 2.1 Study Registration

This systematic review and meta‐analysis was reported in accordance with the guidelines for reporting systematic reviews and meta‐analyses. The study was prospectively registered on the International Prospective Register of Systematic Reviews (PROSPERO) (CRD420251071015).

### 2.2 Eligibility Criteria

#### 2.2.1 Inclusion Criteria

1. Studies enrolling adults (≥ 18 years) with acute spontaneous ICH verified by computed tomography (CT) or magnetic resonance imaging (MRI);
2. Studies which developed or validated ML models designed to predict at least one of the following prespecified poor outcomes: HE, poor functional outcome (defined as mRS 3–6 or equivalent), or all‐cause mortality within 12 months;
3. Studies published in English.

#### 2.2.2 Exclusion Criteria

1. Studies involving subdural, epidural, subarachnoid, traumatic, tumor‐related, or pediatric (age < 18 years) hemorrhage populations;
2. Studies that performed only risk‐factor analysis without developing or validating ML‐based prediction models;
3. Studies lacking performance metrics necessary for quantitative assessment of model accuracy, such as area under the curve (AUC), concordance index (C‐index), sensitivity, specificity, calibration curves, confusion matrices, or F1 score;
4. Studies in which the models focused solely on imaging segmentation, infarct prediction, or outcomes other than HE, functional outcome, or mortality;
5. Studies with no full text available, including conference abstracts, editorials, narrative reviews, study protocols, or duplicate publications based on the same cohort;

### 2.3 Data Sources and Search Strategy

We systematically searched PubMed, Embase, Web of Science, and the Cochrane Library from their inception to September 25, 2025, without restrictions on language, publication date, or geographic region. Each database was searched with a sensitive strategy combining Medical Subject Headings (MeSH) or Emtree terms with free‐text words, including “intracerebral hemorrhage,” “machine learning,” and “prognostic performance” (File S1). The reference lists of all eligible articles and related reviews were hand‐searched to identify additional studies.

### 2.4 Study Selection

All records identified by the search were imported into EndNote 21, and exact duplicates were removed. Two reviewers (DQ and HR) independently screened titles and abstracts against the predefined eligibility criteria, flagging any citation that potentially described an ML model developed or validated for predicting HE, poor functional outcome, or mortality after ICH. The full texts of all potentially eligible articles were obtained and reassessed in duplicate; disagreements were resolved by consensus, with adjudication by a third senior investigator (ZWL) when necessary.

### 2.5 Data Extraction

Prior to data collection, a standardized electronic extraction form was developed and pilot‐tested on five randomly selected articles. The form captured the following information: (1) bibliographic details, including title, first author, year of publication, DOI, and country of origin; (2) study characteristics, including study design, patient source, sample size, number of events, and follow‐up duration; (3) participant characteristics, including ICH subtype, treatment background, diagnostic criteria for the predicted event, event definition, and measurement method; (4) modeling pipeline, including segmentation approach, imaging protocol, segmentation software for regions of interest, and number of radiologists; (5) methodology, including missing‐value handling, feature‐selection strategy, algorithm class, type of predictors, and methods for overfitting evaluation; and (6) model performance metrics. Two reviewers (DQ and LH) independently extracted all data; discrepancies were resolved by consensus and, when necessary, arbitration by a third senior reviewer (ZWL).

### 2.6 Risk of Bias in Studies

The risk of bias in the included studies was assessed using the Prediction model Risk Of Bias ASsessment Tool (PROBAST) (de Jong et al. 2021). PROBAST consists of a series of questions covering participants, predictors, outcomes, and statistical analysis. The four domains include two, three, six, and nine specific questions, respectively, each with three possible responses (yes/probably yes, no/probably no, and no information). A domain was considered to be at high risk of bias if at least one question was answered no/probably no. Conversely, a domain was deemed to be at low risk of bias if all questions were answered as yes/probably yes. Two reviewers (DQ and YY) independently evaluated the risk of bias using PROBAST and cross‐checked their assessments. Any disagreements were resolved by a third reviewer (ZWL).

### 2.7 Outcome Definitions

The three primary outcomes of interest were HE, poor functional outcome, and all‐cause mortality.

HE was most commonly defined as an absolute increase in intracerebral hematoma volume ≥ 6 mL or a relative increase ≥ 33% on follow‐up NCCT compared with the baseline scan. The follow‐up imaging was typically performed within 24–72 h after symptom onset, with 24‐h reassessment being the most prevalent. Some studies adopted alternative definitions, such as an absolute increase of ≥ 12.5 mL.

Poor functional outcome was predominantly assessed using the mRS. Most studies defined poor functional outcome as mRS score 3–6 (moderate‐to‐severe disability or death), while a substantial minority used the more stringent threshold of mRS 4–6 (severe disability or death). The assessment timepoint varied: 90 days post‑hemorrhage was the most common, but other timepoints included at discharge, 30 days, 6 months, and 12 months. A small number of studies used the Glasgow Outcome Scale (GOS), with poor functional outcome defined as GOS 1–3 (death, vegetative state, or severe disability).

Mortality was defined as all‑cause death. The reported assessment timepoints included in‑hospital mortality, 30‑day mortality, 90‑day mortality, 6‑month mortality, and 12‑month mortality.

### 2.8 Synthesis Methods

Meta‐analysis of the C‐index was performed to assess the overall accuracy of ML models in predicting adverse outcomes after ICH. For studies where the 95% confidence interval (CI) and standard error of the C‐index were not reported, the standard error was estimated using the methodology described by Debray et al. (2019). When significant heterogeneity was detected (*I* ^2^ > 50%), a random‐effects model was employed for the meta‐analysis.

Where studies directly reported sensitivity, specificity, and sample sizes, these were used to calculate summary estimates of sensitivity and specificity. Given the limited reporting of complete paired sensitivity and specificity data in the original studies, a formal bivariate meta‐analysis based on reconstructed diagnostic 2 × 2 tables was not performed, as such reconstruction would introduce additional uncertainty that could not be adequately quantified. This limitation in the original studies precluded a robust pooled analysis of calibration‐related metrics.

Publication bias was assessed for meta‐analyses that included at least 10 studies. Potential small‐study effects were evaluated through visual inspection of funnel plots and Egger's linear regression test, with a two‐sided *p* value < 0.10 considered indicative of significant asymmetry.

## 3 Results

### 3.1 Study Selection

**FIGURE 1:**
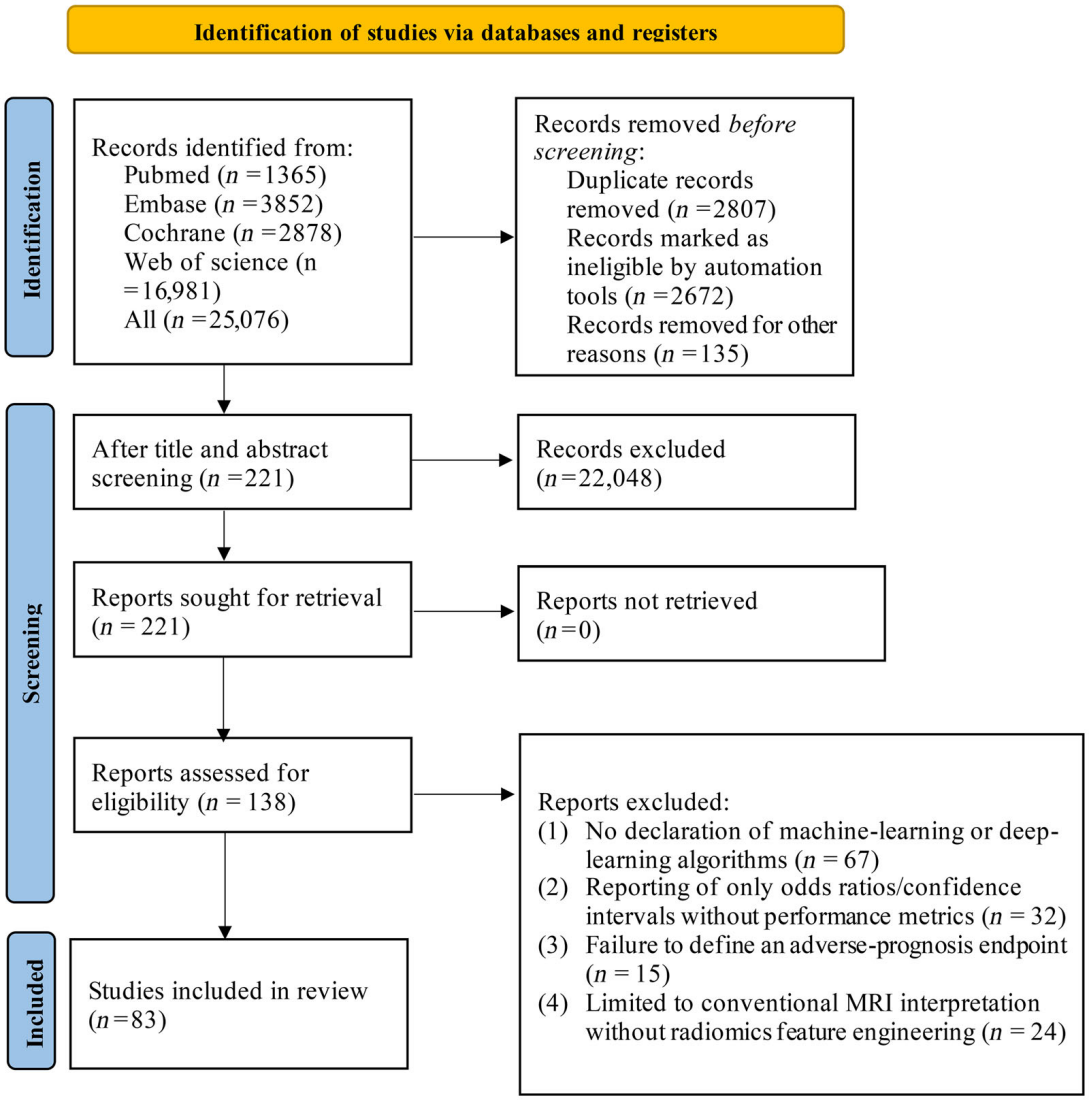
Flowchart of the literature search and selection process.

The comprehensive search yielded 25,076 records from PubMed (*n* = 1365), Embase (*n* = 3852), Web of Science (*n* = 16,981), and Cochrane Library (*n* = 2878). After removing 2807 duplicates, including 2672 automatically by EndNote and 135 manually, 22,269 records remained. After the screening of titles and abstracts, 22,048 records were excluded due to failure to meet the eligibility criteria, leaving 221 articles for full‐text assessment. Following the full‐text review of the remaining 221 articles, 138 articles were eliminated, with 67 for the absence of ML or DL algorithms, 32 for reporting only odds ratios or CIs without any performance measures (e.g., AUC), 15 for failure to define an adverse outcome, and 24 for limitation to conventional MRI interpretation without radiomics features. Consequently, 83 unique studies (Bo et al. 2023; Q. Chen et al. 2024; Q. Chen, Zhu, et al. 2021; W. Chen, Li, et al. 2021; Y. Chen, Jiang, et al. 2023; Y. Chen, Liu, et al. 2025; Y. Chen, Rivier, et al. 2025; Z. F. Chen, Zhang, et al. 2023; Dierksen et al. 2024; Duan et al. 2022; Q. Feng et al. 2023; Gao et al. 2023, 2022; Geng et al. 2024; Guo et al. 2022; Gupta et al. 2017; Haider et al. 2023; Hall et al. 2021; Hegde et al. 2024; Hu et al. 2024; Huang et al. 2023, 2022; Hung et al. 2023; H. Li et al. 2022; Liang et al. 2025; Lim et al. 2022, 2024; Ling et al. 2024; López‐Rueda et al. 2024; Lukić et al. 2012; Luo et al. 2024; Mao et al. 2024; Matsumoto et al. 2024; Menon et al. 2022; Misra et al. 2024; Nawabi et al. 2021; Nie et al. 2020; Pan et al. 2024; Pei et al. 2024; S. Y. Peng et al. 2010; Pérez Del Barrio et al. 2023; Phan et al. 2017; Pszczolkowski et al. 2021; Qi et al. 2022; Seymour et al. 2022; Shi et al. 2024; X. Song et al. 2024; Z. Song, Guo, et al. 2021; Z. Song, Tang, et al. 2021; Sonobe et al. 2022; Takahashi et al. 2006; Tang et al. 2022; Trevisi et al. 2022; D. Wang, Zhang, Dong, et al. 2025; H. L. Wang et al. 2019; J. Wang, Zhou, et al. 2023; K. Wang, Liu, et al. 2024; M. Wang, Liang, et al. 2024; Y. Wang, Deng, et al. 2023; Q. Wu and Gao 2025; T. C. Wu et al. 2023, 2022; Xia et al. 2025, 2022, 2023; H. Xie et al. 2020; Y. Xie et al. 2022; Xing et al. 2023; M. Xu et al. 2024; W. Xu et al. 2022; X. Xu et al. 2021; Yalcin et al. 2024; Yang et al. 2021; Yap et al. 2025; Ye et al. 2024; Yeo et al. 2025; Yu et al. 2025; Zhan et al. 2021; H. Zhang et al. 2024; K. Zhang et al. 2023; Zhou et al. 2022, 2024, 2021) fulfilled all eligibility requirements and were incorporated into the qualitative and quantitative synthesis (Figure 1).

### 3.2 Study Characteristics

A total of 83 studies were included in this systematic review and meta‐analysis. The characteristics of these studies are described below, categorized into clinical feature‐based models and radiomics‐based ML models.

#### 3.2.1 Clinical Feature‐Based Models

A total of 53 studies explored clinical feature‐based models (Bo et al. 2023; Q. Chen et al. 2024; W. Chen, Li, et al. 2021; Y. Chen, Jiang, et al. 2023; Y. Chen, Liu, et al. 2025; Y. Chen, Rivier, et al. 2025; Dierksen et al. 2024; Duan et al. 2022; Q. Feng et al. 2023; Gao et al. 2023, 2022; Geng et al. 2024; Guo et al. 2022; Gupta et al. 2017; Hall et al. 2021; Hegde et al. 2024; Hu et al. 2024; Hung et al. 2023; Lim et al. 2022, 2024; Ling et al. 2024; Lukić et al. 2012; Mao et al. 2024; Matsumoto et al. 2024; Menon et al. 2022; Misra et al. 2024; Nawabi et al. 2021; Nie et al. 2020; Pan et al. 2024; S. Y. Peng et al. 2010; Pérez Del Barrio et al. 2023; Phan et al. 2017; Qi et al. 2022; Shi et al. 2024; Sonobe et al. 2022; Takahashi et al. 2006; Tang et al. 2022; Trevisi et al. 2022; D. Wang, Zhang, Dong, et al. 2025; H. L. Wang et al. 2019; K. Wang, Liu, et al. 2024; Y. Wang, Deng, et al. 2023; Q. Wu and Gao 2025; Xia et al. 2023; Xing et al. 2023; M. Xu et al. 2024; Yalcin et al. 2024; Yang et al. 2021; Yap et al. 2025; Ye et al. 2024; Yeo et al. 2025; H. Zhang et al. 2024; K. Zhang et al. 2023). These studies were published between 2006 and 2025, with the publication number increasing markedly after 2019 (Bo et al. 2023; Q. Chen et al. 2024; W. Chen, Li, et al. 2021; Y. Chen, Jiang, et al. 2023; Y. Chen, Liu, et al. 2025; Y. Chen, Rivier, et al. 2025; Dierksen et al. 2024; Duan et al. 2022; Q. Feng et al. 2023; Gao et al. 2023, 2022; Geng et al. 2024; Guo et al. 2022; Hall et al. 2021; Hegde et al. 2024; Hu et al. 2024; Hung et al. 2023; Lim et al. 2022, 2024; Ling et al. 2024; Mao et al. 2024; Matsumoto et al. 2024; Menon et al. 2022; Misra et al. 2024; Nawabi et al. 2021; Nie et al. 2020; Pan et al. 2024; Pérez Del Barrio et al. 2023; Qi et al. 2022; Shi et al. 2024; Sonobe et al. 2022; Tang et al. 2022; Trevisi et al. 2022; D. Wang, Zhang, Dong, et al. 2025; K. Wang, Liu, et al. 2024; Y. Wang, Deng, et al. 2023; Q. Wu and Gao 2025; Xia et al. 2023; Xing et al. 2023; M. Xu et al. 2024; Yalcin et al. 2024; Yang et al. 2021; Yap et al. 2025; Ye et al. 2024; Yeo et al. 2025; H. Zhang et al. 2024; K. Zhang et al. 2023) (*n* = 47). The research was conducted across 15 countries, primarily in China (Bo et al. 2023; Q. Chen et al. 2024; W. Chen, Li, et al. 2021; Y. Chen, Jiang, et al. 2023; Y. Chen, Liu, et al. 2025; Duan et al. 2022; Gao et al. 2023, 2022; Geng et al. 2024; Guo et al. 2022; Hu et al. 2024; Hung et al. 2023; Mao et al. 2024; Nie et al. 2020; Pan et al. 2024; S. Y. Peng et al. 2010; Qi et al. 2022; Shi et al. 2024; Tang et al. 2022; D. Wang, Zhang, Dong, et al. 2025; H. L. Wang et al. 2019; K. Wang, Liu, et al. 2024; Y. Wang, Deng, et al. 2023; Q. Wu and Gao 2025; Xia et al. 2023; Xing et al. 2023; M. Xu et al. 2024; Yang et al. 2021; Yap et al. 2025; Ye et al. 2024; H. Zhang et al. 2024; K. Zhang et al. 2023) (*n* = 32), the United States (W. Chen, Li, et al. 2021; Y. Chen, Rivier, et al. 2025; Dierksen et al. 2024; Q. Feng et al. 2023; Gupta et al. 2017; Hall et al. 2021; Hegde et al. 2024; Ling et al. 2024; Misra et al. 2024; Phan et al. 2017; Takahashi et al. 2006) (*n* = 11), Japan (Matsumoto et al. 2024; Sonobe et al. 2022; Takahashi et al. 2006) (*n* = 3), and Singapore (Lim et al. 2022, 2024; Yeo et al. 2025) (*n* = 3), among others.

Among the studies, 29 were retrospective cohort studies (W. Chen, Li, et al. 2021; Y. Chen, Jiang, et al. 2023; Dierksen et al. 2024; Gao et al. 2023; Guo et al. 2022; Gupta et al. 2017; Hall et al. 2021; Hegde et al. 2024; Hu et al. 2024; Hung et al. 2023; Lim et al. 2022, 2024; Ling et al. 2024; Mao et al. 2024; Matsumoto et al. 2024; Menon et al. 2022; Nawabi et al. 2021; Pan et al. 2024; Phan et al. 2017; Sonobe et al. 2022; Takahashi et al. 2006; Trevisi et al. 2022; D. Wang, Zhang, Dong, et al. 2025; Y. Wang, Deng, et al. 2023; Xia et al. 2023; Yang et al. 2021; Ye et al. 2024; Yeo et al. 2025; K. Zhang et al. 2023), four were prospective cohort studies (Y. Chen, Rivier, et al. 2025; Lukić et al. 2012; Misra et al. 2024; K. Wang, Liu, et al. 2024), and 20 were case‐control studies (Bo et al. 2023; Q. Chen et al. 2024; Y. Chen, Liu, et al. 2025; Duan et al. 2022; Q. Feng et al. 2023; Gao et al. 2022; Geng et al. 2024; Nie et al. 2020; S. Y. Peng et al. 2010; Pérez Del Barrio et al. 2023; Qi et al. 2022; Shi et al. 2024; Tang et al. 2022; H. L. Wang et al. 2019; Q. Wu and Gao 2025; Xing et al. 2023; M. Xu et al. 2024; Yalcin et al. 2024; Yap et al. 2025; H. Zhang et al. 2024). Meanwhile, 30 studies adopted single‐center designs (Bo et al. 2023; W. Chen, Li, et al. 2021; Duan et al. 2022; Guo et al. 2022; Gupta et al. 2017; Hegde et al. 2024; Hung et al. 2023; Lim et al. 2022, 2024; Lukić et al. 2012; Matsumoto et al. 2024; Menon et al. 2022; Misra et al. 2024; S. Y. Peng et al. 2010; Pérez Del Barrio et al. 2023; Qi et al. 2022; Shi et al. 2024; Sonobe et al. 2022; Takahashi et al. 2006; Tang et al. 2022; H. L. Wang et al. 2019; Y. Wang, Deng, et al. 2023; Q. Wu and Gao 2025; Xing et al. 2023; M. Xu et al. 2024; Yalcin et al. 2024; Yang et al. 2021; Yap et al. 2025; Ye et al. 2024; Yeo et al. 2025), 20 studies adopted multicenter designs (Q. Chen et al. 2024; Y. Chen, Jiang, et al. 2023; Y. Chen, Rivier, et al. 2025; Dierksen et al. 2024; Gao et al. 2023, 2022; Geng et al. 2024; Hall et al. 2021; Hu et al. 2024; Ling et al. 2024; Mao et al. 2024; Nawabi et al. 2021; Pan et al. 2024; Phan et al. 2017; Trevisi et al. 2022; D. Wang, Zhang, Dong, et al. 2025; K. Wang, Liu, et al. 2024; Xia et al. 2023; H. Zhang et al. 2024; K. Zhang et al. 2023), and an additional three studies employed the Medical Information Mart for Intensive Care database (Y. Chen, Liu, et al. 2025; Q. Feng et al. 2023; Nie et al. 2020). All studies exclusively involved patients with spontaneous ICH.

The primary outcome events were poor functional outcome (e.g., mRS 3–6 or GOS 1–3), assessed in 32 studies (Y. Chen, Jiang, et al. 2023; Y. Chen, Rivier, et al. 2025; Dierksen et al. 2024; Gao et al. 2023; Geng et al. 2024; Guo et al. 2022; Gupta et al. 2017; Hall et al. 2021; Hegde et al. 2024; Hu et al. 2024; Hung et al. 2023; Lim et al. 2022, 2024; Ling et al. 2024; Matsumoto et al. 2024; Menon et al. 2022; Misra et al. 2024; Nawabi et al. 2021; Pan et al. 2024; Phan et al. 2017; Qi et al. 2022; Sonobe et al. 2022; D. Wang, Zhang, Dong, et al. 2025; H. L. Wang et al. 2019; K. Wang, Liu, et al. 2024; Q. Wu and Gao 2025; Xia et al. 2023; Xing et al. 2023; M. Xu et al. 2024; Yang et al. 2021; H. Zhang et al. 2024; K. Zhang et al. 2023), and mortality, assessed in 13 studies (W. Chen, Li, et al. 2021; Y. Chen, Liu, et al. 2025; Q. Feng et al. 2023; Lukić et al. 2012; Nie et al. 2020; S. Y. Peng et al. 2010; Pérez Del Barrio et al. 2023; Shi et al. 2024; Takahashi et al. 2006; Y. Wang, Deng, et al. 2023; Yang et al. 2021; Yap et al. 2025; Yeo et al. 2025). HE or neurological deterioration was assessed in 11 studies (Bo et al. 2023; Q. Chen et al. 2024; Duan et al. 2022; Gao et al. 2022; Mao et al. 2024; Matsumoto et al. 2024; Tang et al. 2022; Trevisi et al. 2022; Xing et al. 2023; Yalcin et al. 2024; Ye et al. 2024). The cumulative sample size across these studies exceeded 104,900 patients.

Model validation was performed using internal methods (e.g., cross‐validation) in 39 studies (Bo et al. 2023; Q. Chen et al. 2024; W. Chen, Li, et al. 2021; Y. Chen, Jiang, et al. 2023; Duan et al. 2022; Q. Feng et al. 2023; Gao et al. 2023, 2022; Guo et al. 2022; Hegde et al. 2024; Hung et al. 2023; Lim et al. 2022, 2024; Ling et al. 2024; Lukić et al. 2012; Menon et al. 2022; Misra et al. 2024; Nawabi et al. 2021; Nie et al. 2020; Pan et al. 2024; S. Y. Peng et al. 2010; Pérez Del Barrio et al. 2023; Phan et al. 2017; Qi et al. 2022; Shi et al. 2024; Sonobe et al. 2022; Tang et al. 2022; Trevisi et al. 2022; D. Wang, Zhang, Dong, et al. 2025; H. L. Wang et al. 2019; K. Wang, Liu, et al. 2024; Y. Wang, Deng, et al. 2023; Q. Wu and Gao 2025; Xing et al. 2023; M. Xu et al. 2024; Yalcin et al. 2024; Yap et al. 2025; Ye et al. 2024; Yeo et al. 2025), external validation in five studies (Dierksen et al. 2024; Gupta et al. 2017; Matsumoto et al. 2024; Xia et al. 2023; H. Zhang et al. 2024), and a combination of both internal and external validation in seven studies (Y. Chen, Liu, et al. 2025; Y. Chen, Rivier, et al. 2025; Geng et al. 2024; Hall et al. 2021; Hu et al. 2024; Mao et al. 2024; K. Zhang et al. 2023) (Table S1).

#### 3.2.2 Radiomics‐Based Machine Learning Models

A total of 30 studies developed radiomics‐based ML models (Q. Chen, Zhu, et al. 2021; Z. F. Chen, Zhang, et al. 2023; Haider et al. 2023; Huang et al. 2023, 2022; H. Li et al. 2022; Liang et al. 2025; López‐Rueda et al. 2024; Luo et al. 2024; Pei et al. 2024; Pszczolkowski et al. 2021; Seymour et al. 2022; X. Song et al. 2024; Z. Song, Guo, et al. 2021; Z. Song, Tang, et al. 2021; J. Wang, Zhou, et al. 2023; M. Wang, Liang, et al. 2024; T. C. Wu et al. 2023, 2022; Xia et al. 2025, 2022; H. Xie et al. 2020; Y. Xie et al. 2022; W. Xu et al. 2022; X. Xu et al. 2021; Yu et al. 2025; Zhan et al. 2021; Zhou et al. 2022, 2024, 2021). These studies were published between 2019 and 2025, reflecting the more recent application of radiomics in this field. They were conducted in several countries, with a strong predominance of studies from China (Q. Chen, Zhu, et al. 2021; Huang et al. 2023, 2022; H. Li et al. 2022; Liang et al. 2025; Luo et al. 2024; Pei et al. 2024; X. Song et al. 2024; Z. Song, Guo, et al. 2021; Z. Song, Tang, et al. 2021; J. Wang, Zhou, et al. 2023; M. Wang, Liang, et al. 2024; T. C. Wu et al. 2023, 2022; Xia et al. 2025, 2022; H. Xie et al. 2020; Y. Xie et al. 2022; W. Xu et al. 2022; X. Xu et al. 2021; Yu et al. 2025; Zhan et al. 2021; Zhou et al. 2022, 2024, 2021) (*n* = 25), followed by the United States (Z. F. Chen, Zhang, et al. 2023; Haider et al. 2023; Seymour et al. 2022; T. C. Wu et al. 2023, 2022) (*n* = 5), the United Kingdom (Haider et al. 2023; Pszczolkowski et al. 2021) (*n* = 2), Spain (López‐Rueda et al. 2024) (*n* = 1), and Malaysia (Pszczolkowski et al. 2021) (*n* = 1).

The included studies were predominantly retrospective cohort investigations (*n* = 17) (Q. Chen, Zhu, et al. 2021; Z. F. Chen, Zhang, et al. 2023; Huang et al. 2023, 2022; Liang et al. 2025; López‐Rueda et al. 2024; Luo et al. 2024; Pei et al. 2024; Pszczolkowski et al. 2021; Z. Song, Tang, et al. 2021; J. Wang, Zhou, et al. 2023; H. Xie et al. 2020; X. Xu et al. 2021; Zhan et al. 2021; Zhou et al. 2022, 2024, 2021), followed by case‐control designs (*n* = 10) (H. Li et al. 2022; Seymour et al. 2022; X. Song et al. 2024; Z. Song, Guo, et al. 2021; T. C. Wu et al. 2023, 2022; Xia et al. 2025, 2022; W. Xu et al. 2022; Yu et al. 2025). Two reports utilized combined retrospective and prospective cohort approaches (M. Wang, Liang, et al. 2024; Y. Xie et al. 2022), and one was a purely prospective cohort study (Haider et al. 2023). Patient sources included 14 single‐center studies (Q. Chen, Zhu, et al. 2021; H. Li et al. 2022; López‐Rueda et al. 2024; Luo et al. 2024; Pei et al. 2024; Seymour et al. 2022; X. Song et al. 2024; Z. Song, Guo, et al. 2021; T. C. Wu et al. 2023, 2022; H. Xie et al. 2020; W. Xu et al. 2022; X. Xu et al. 2021; Zhan et al. 2021) and 16 multicenter studies (Z. F. Chen, Zhang, et al. 2023; Haider et al. 2023; Huang et al. 2023, 2022; Liang et al. 2025; Pszczolkowski et al. 2021; Z. Song, Tang, et al. 2021; J. Wang, Zhou, et al. 2023; M. Wang, Liang, et al. 2024; Xia et al. 2025, 2022; Y. Xie et al. 2022; Yu et al. 2025; Zhou et al. 2022, 2024, 2021). All studies involved spontaneous ICH, and the source of radiomics features was exclusively NCCT.

The outcome events were mainly poor functional outcome (mRS 3–6 or 4–6), investigated in 14 studies (Huang et al. 2023, 2022; Liang et al. 2025; Pei et al. 2024; Pszczolkowski et al. 2021; X. Song et al. 2024; Z. Song, Tang, et al. 2021; J. Wang, Zhou, et al. 2023; T. C. Wu et al. 2023; Xia et al. 2025; Y. Xie et al. 2022; X. Xu et al. 2021; Zhou et al. 2022, 2021), and HE, investigated in 13 studies (Q. Chen, Zhu, et al. 2021; Z. F. Chen, Zhang, et al. 2023; Haider et al. 2023; H. Li et al. 2022; Seymour et al. 2022; Z. Song, Guo, et al. 2021; M. Wang, Liang, et al. 2024; T. C. Wu et al. 2022; Xia et al. 2022; H. Xie et al. 2020; W. Xu et al. 2022; Yu et al. 2025; Zhou et al. 2024). One study focused on mortality (López‐Rueda et al. 2024). The cumulative sample size encompassed over 31,940 patients. Model validation strategies included internal validation in 15 studies (Q. Chen, Zhu, et al. 2021; Z. F. Chen, Zhang, et al. 2023; H. Li et al. 2022; López‐Rueda et al. 2024; Luo et al. 2024; Pei et al. 2024; Pszczolkowski et al. 2021; Seymour et al. 2022; X. Song et al. 2024; Z. Song, Guo, et al. 2021; T. C. Wu et al. 2023, 2022; H. Xie et al. 2020; X. Xu et al. 2021; Zhan et al. 2021), external validation in six studies (Haider et al. 2023; Liang et al. 2025; Xia et al. 2025; W. Xu et al. 2022; Zhou et al. 2024, 2021), and a combination of both internal and external validation in nine studies (Huang et al. 2023, 2022; Z. Song, Tang, et al. 2021; J. Wang, Zhou, et al. 2023; M. Wang, Liang, et al. 2024; Xia et al. 2022; Y. Xie et al. 2022; Yu et al. 2025; Zhou et al. 2022).

In summary, the included literature demonstrated a comprehensive exploration of both clinical and radiomics‐based predictors for outcomes following ICH, utilizing diverse methodologies and validation approaches (Table S1). It should be noted that only a small minority of the included studies reported any formal model calibration metrics (e.g., Hosmer–Lemeshow test, calibration‐in‐the‐large, calibration plots), and decision curve analysis to evaluate net clinical benefit was absent in almost all studies (Table S1).

### 3.3 Risk of Bias in Studies

**FIGURE 2:**
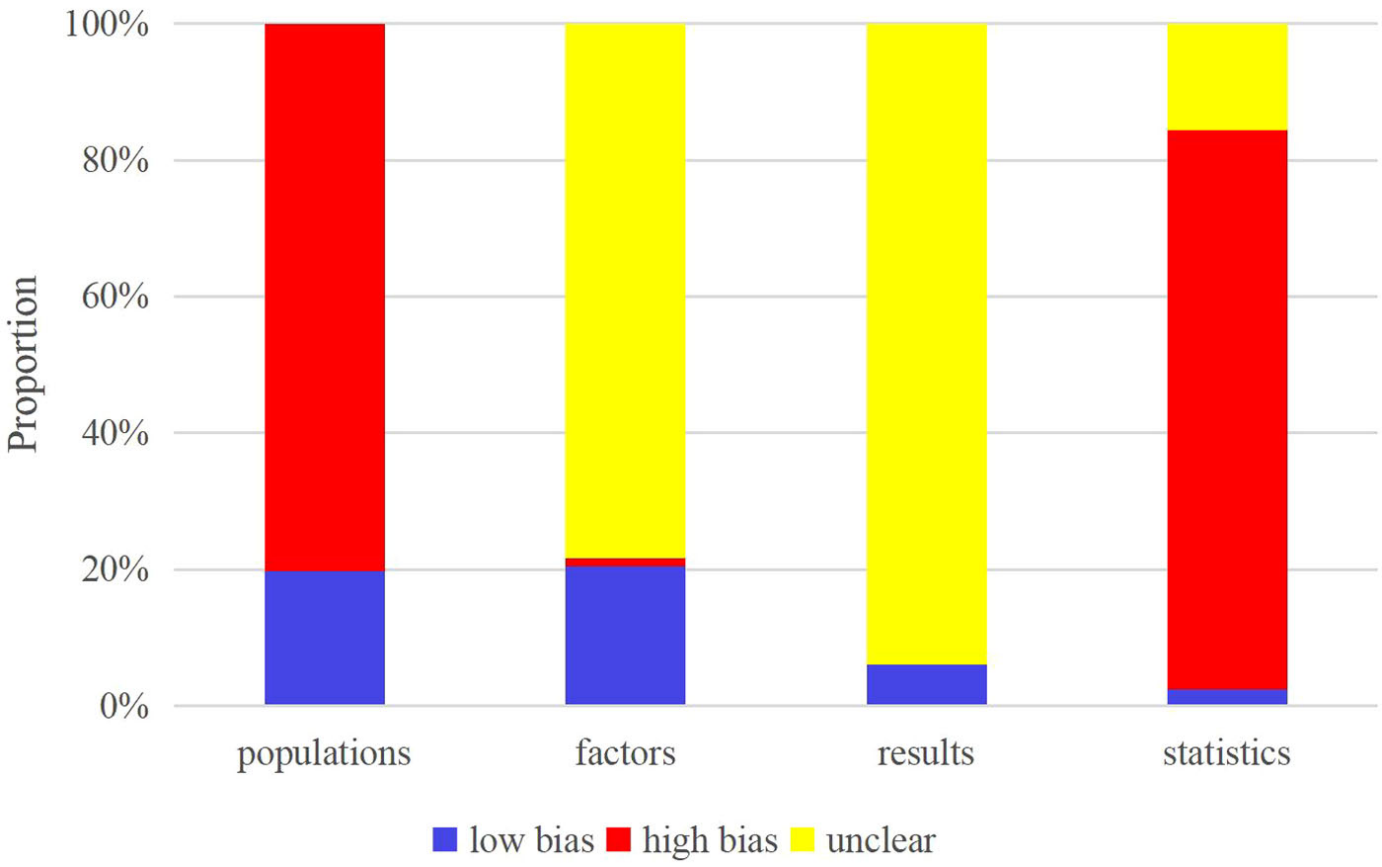
Results of PROBAST assessment for included studies.

The risk of bias assessment for the 83 included studies revealed an overall high risk, predominantly attributable to their retrospective or case‐control design (Bo et al. 2023; Q. Chen et al. 2024; Q. Chen, Zhu, et al. 2021; W. Chen, Li, et al. 2021; Y. Chen, Jiang, et al. 2023; Z. F. Chen, Zhang, et al. 2023; Dierksen et al. 2024; Duan et al. 2022; Gao et al. 2023, 2022; Geng et al. 2024; Guo et al. 2022; Haider et al. 2023; Hu et al. 2024; Huang et al. 2023, 2022; H. Li et al. 2022; Liang et al. 2025; Lim et al. 2022, 2024; López‐Rueda et al. 2024; Luo et al. 2024; Mao et al. 2024; Matsumoto et al. 2024; Nawabi et al. 2021; Nie et al. 2020; Pan et al. 2024; Pei et al. 2024; S. Y. Peng et al. 2010; Pérez Del Barrio et al. 2023; Pszczolkowski et al. 2021; Qi et al. 2022; Seymour et al. 2022; Shi et al. 2024; X. Song et al. 2024; Z. Song, Guo, et al. 2021; Z. Song, Tang, et al. 2021; Sonobe et al. 2022; Takahashi et al. 2006; Tang et al. 2022; Trevisi et al. 2022; D. Wang, Zhang, Dong, et al. 2025; H. L. Wang et al. 2019; J. Wang, Zhou, et al. 2023; M. Wang, Liang, et al. 2024; T. C. Wu et al. 2023, 2022; Xia et al. 2025, 2022, 2023; H. Xie et al. 2020; Y. Xie et al. 2022; Xing et al. 2023; M. Xu et al. 2024; W. Xu et al. 2022; X. Xu et al. 2021; Yalcin et al. 2024; Yap et al. 2025; Ye et al. 2024; Yeo et al. 2025; Yu et al. 2025; Zhan et al. 2021; H. Zhang et al. 2024; K. Zhang et al. 2023; Zhou et al. 2022, 2024, 2021), which inherently introduces selection bias. Although the inclusion and exclusion criteria were generally well‐defined and appropriate across studies, the nature of the data source constituted the primary concern in the study population domain. Regarding predictors, while most studies applied consistent definitions and assessment methods, a substantial number failed to report whether predictor assessment was performed blinded to outcome information (Bo et al. 2023; Q. Chen et al. 2024; Q. Chen, Zhu, et al. 2021; W. Chen, Li, et al. 2021; Y. Chen, Jiang, et al. 2023; Y. Chen, Liu, et al. 2025; Y. Chen, Rivier, et al. 2025; Dierksen et al. 2024; Q. Feng et al. 2023; Gao et al. 2023, 2022; Geng et al. 2024; Guo et al. 2022; Gupta et al. 2017; Haider et al. 2023; Hegde et al. 2024; Hu et al. 2024; Huang et al. 2023, 2022; H. Li et al. 2022; Liang et al. 2025; Lim et al. 2022, 2024; Ling et al. 2024; Lukić et al. 2012; Luo et al. 2024; Mao et al. 2024; Matsumoto et al. 2024; Menon et al. 2022; Nawabi et al. 2021; Nie et al. 2020; S. Y. Peng et al. 2010; Pérez Del Barrio et al. 2023; Phan et al. 2017; Qi et al. 2022; Seymour et al. 2022; X. Song et al. 2024; Z. Song, Guo, et al. 2021; Z. Song, Tang, et al. 2021; Takahashi et al. 2006; Tang et al. 2022; Trevisi et al. 2022; D. Wang, Zhang, Dong, et al. 2025; H. L. Wang et al. 2019; J. Wang, Zhou, et al. 2023; K. Wang, Liu, et al. 2024; M. Wang, Liang, et al. 2024; Q. Wu and Gao 2025; T. C. Wu et al. 2023, 2022; Xia et al. 2025, 2022, 2023; H. Xie et al. 2020; Y. Xie et al. 2022; Xing et al. 2023; M. Xu et al. 2024; W. Xu et al. 2022; X. Xu et al. 2021; Yalcin et al. 2024; Yang et al. 2021; Yap et al. 2025; Ye et al. 2024; Yeo et al. 2025; Yu et al. 2025; Zhan et al. 2021; Zhou et al. 2022, 2024, 2021), resulting in an unclear risk of bias. Furthermore, the use of univariate analysis for predictor selection in several studies (Guo et al. 2022; Hegde et al. 2024; Huang et al. 2023, 2022; Lim et al. 2022; Lukić et al. 2012; Luo et al. 2024; Misra et al. 2024; Pei et al. 2024; Shi et al. 2024; X. Song et al. 2024; Z. Song, Guo, et al. 2021; Z. Song, Tang, et al. 2021; Takahashi et al. 2006; D. Wang, Zhang, Dong, et al. 2025; H. L. Wang et al. 2019; J. Wang, Zhou, et al. 2023; M. Wang, Liang, et al. 2024; Xia et al. 2025, 2022, 2023; H. Xie et al. 2020; Y. Xie et al. 2022; Xing et al. 2023; W. Xu et al. 2022; X. Xu et al. 2021; Yang et al. 2021; Yeo et al. 2025; Yu et al. 2025; Zhan et al. 2021; H. Zhang et al. 2024; Zhou et al. 2022, 2021) introduced a high risk of overfitting and selection bias. In contrast, the outcome domain was consistently at low risk, as outcomes such as HE and functional outcome (e.g., mRS) were objectively defined using standard criteria and prespecified. For statistical analysis, a high risk of bias frequently arose from insufficient sample size, evidenced by an events‐per‐variable ratio below 10 in numerous studies (Q. Chen et al. 2024; Gao et al. 2023; Guo et al. 2022; Hu et al. 2024; López‐Rueda et al. 2024; Luo et al. 2024; Nawabi et al. 2021; Nie et al. 2020; Pan et al. 2024; Pei et al. 2024; Pérez Del Barrio et al. 2023; Qi et al. 2022; Shi et al. 2024; X. Song et al. 2024; Sonobe et al. 2022; Tang et al. 2022; H. L. Wang et al. 2019; Q. Wu and Gao 2025; T. C. Wu et al. 2023, 2022; Xia et al. 2022; H. Xie et al. 2020; Xing et al. 2023; M. Xu et al. 2024; X. Xu et al. 2021; Yalcin et al. 2024; K. Zhang et al. 2023), coupled with the absence of robust independent validation or the use of validation cohorts with fewer than 100 participants (Q. Chen et al. 2024; Duan et al. 2022; Gao et al. 2023; Hu et al. 2024; López‐Rueda et al. 2024; Luo et al. 2024; Nawabi et al. 2021; Nie et al. 2020; Pan et al. 2024; Pei et al. 2024; Qi et al. 2022; X. Song et al. 2024; Sonobe et al. 2022; Tang et al. 2022; T. C. Wu et al. 2023, 2022; Xia et al. 2022, 2023; H. Xie et al. 2020; Xing et al. 2023; X. Xu et al. 2021; K. Zhang et al. 2023), thereby limiting the generalizability and stability of the developed models (Figure 2).

### 3.4 Meta‐Analysis

Given the anticipated clinical and methodological heterogeneity across studies, all meta‐analyses were stratified by the three prespecified outcome domains (HE, poor functional outcome, and mortality). Within each domain, pooled estimates were further stratified by predictor category (clinical, radiomics, and combined clinical‐radiomics) and by ML algorithm type to mitigate the influence of these factors on the summary results. The detailed subgroup results are presented below and in Tables 1, 2, 3.

**TABLE 1:** Pooled C‐index of machine learning for predicting hematoma expansion.

Subgroup analysis	Model	Training set			Validation set		
		*n*	C‐index (95% CI)	*I* ^2^ (%)	*n*	C‐index (95% CI)	*I* ^2^ (%)
Clinical							
	LR	6	0.751 (0.714–0.788)			0.712 (0.674–0.751)	
Overall			0.751 (0.714–0.788)			0.712 (0.674–0.751)	
Radiomics							
	LR	11	0.876 (0.838–0.915)	91.3	13	0.811 (0.775–0.847)	65.5
	RM	1	0.808 (0.755–0.861)		1	0.739 (0.670–0.808)	
	ANN	1	0.823 (0.741–0.905)		1	0.823 (0.696–0.950)	
	DT	4	0.805 (0.769–0.841)	15.1	4	0.763 (0.710–0.817)	0
	RF	1	0.906 (0.845–0.967)		1	0.906 (0.812–1.000)	
	SVM	5	0.839 (0.780–0.898)	79.6	3	0.757 (0.598–0.916)	87.9
	NB	1	0.861 (0.788–0.934)		1	0.861 (0.748–0.974)	
	KNN	1	0.910 (0.851–0.969)		1	0.910 (0.819–1.001)	
	LASSO	3	0.730 (0.637–0.824)	85.8	3	0.732 (0.598–0.866)	90.1
	AdaBoost	2	0.912 (0.856–0.967)	73.5	2	0.623 (0.552–0.694)	0
Overall		30	0.847 (0.818–0.875)	90.7	30	0.786 (0.754–0.819)	78.1
Radiomics + Clinical							
	LR	11	0.871 (0.830–0.913)	89.1	15	0.826 (0.797–0.856)	55.4
	RF	1	0.850 (0.794–0.906)		1	0.850 (0.794–0.906)	
	SVM	1	0.830 (0.770–0.890)		2	0.891 (0.774–1.007)	88.6
	XGB	1	0.890 (0.841–0.939)		1	0.850 (0.794–0.906)	
	LASSO	2	0.707 (0.619–0.795)	61.8	2	0.697 (0.565–0.828)	75.1
	Nomo	1	0.800 (0.736–0.864)		1	0.760 (0.691–0.829)	
Overall		17	0.847 (0.808–0.885)	90.5	23	0.822 (0.789–0.855)	79

Abbreviations: AdaBoost, Adaptive Boosting; ANN, Artificial Neural Network; CI, confidence interval; DT, Decision Tree; KNN, *K*‐Nearest Neighbors; LASSO, Least Absolute Shrinkage and Selection Operator; LR, logistic regression; NB, Naive Bayes; Nomo, nomogram; RF, Random Forest; RM, radiomics model; SVM, Support Vector Machine; XGB, XGBoost.

**TABLE 2:** Pooled C‐index of machine learning for predicting poor functional outcome.

Subgroup analysis	Model	Training set			Validation set		
		*n*	C‐index (95% CI)	*I* ^2^ (%)	*n*	C‐index (95%CI)	*I* ^2^ (%)
Clinical							
	LR	10	0.841 (0.791–0.890)	95.6	10	0.756 (0.713–0.799)	94.2
	RF	5	0.900 (0.861–0.939)	86.3	6	0.883 (0.828–0.938)	72.8
	ANN				2	0.808 (0.683–0.933)	69.4
	LR		0.841 (0.791–0.890)	95.6			
	DT	1	0.950 (0.920–0.980)		2	0.916 (0.876–0.957)	0
	RF		0.900 (0.861–0.939)	86.3			
	SVM	2	0.739 (0.519–0.960)	95.4	4	0.762 (0.642–0.882)	89
	XGB	2	0.930 (0.803–1.056)	96.3	3	0.896 (0.814–0.977)	91.6
	CatBoost	1	0.871 (0.829–0.913)				
	AutoML	2	0.860 (0.845–0.875)				
	DL				2	0.864 (0.833–0.895)	
	NB				2	0.906 (0.851–0.962)	0
Overall		23	0.861 (0.824–0.899)	98	31	0.822 (0.775–0.868)	97.4
Radiomics							
	LR	3	0.841 (0.816–0.867)	0	11	0.846 (0.807–0.885)	71.9
	RF	4	0.867 (0.778–0.955)	96.4	1	0.830 (0.700–0.960)	
	RM	1	0.727 (0.652–0.802)		4	0.754 (0.709–0.798)	0
	SVM	1	0.800 (0.700–0.900)		4	0.928 (0.887–0.969)	0
	XGB	1	0.920 (0.825–1.015)				
	DL	1	0.910 (0.889–0.931)		3	0.763 (0.671–0.854)	92.3
	KNN				4	0.927 (0.885–0.969)	0
	NB				1	0.839 (0.807–0.871)	
Overall		11	0.855 (0.811–0.899)	92	28		82.8
Radiomics + Clinical							
	LR	16	0.865 (0.843–0.888)	82.9	25	0.848 (0.829–0.868)	47.6
	RF	5	0.887 (0.843–0.932)	94	1	0.860 (0.740–0.980)	
	ANN						
	DT	3	0.774 (0.742–0.806)	75.3	1	0.660 (0.570–0.750)	
	SVM				6	0.894 (0.852–0.936)	87.5
	LASSO		0.823 (0.770–0.876)		2	0.791 (0.740–0.843)	0
	DL				1	0.864 (0.829–0.899)	
Overall			0.856 (0.832–0.880)	93.5	36	0.850 (0.830–0.869)	74.2

Abbreviations: ANN, Artificial Neural Network; AutoML, Automated Machine Learning; CatBoost, Categorical Boosting; CI, confidence interval; DL, deep learning; DT, Decision Tree; KNN, *K*‐Nearest Neighbors; LASSO, Least Absolute Shrinkage and Selection Operator; LR, logistic regression; NB, Naive Bayes; RF, Random Forest; RM, radiomics model; SVM, Support Vector Machine; XGB, XGBoost.

**TABLE 3:** Pooled C‐index of machine learning for predicting mortality.

Subgroup analysis	Model	Training set			Validation set		
		*n*	C‐index (95% CI)	*I* ^2^ (%)	*n*	C‐index (95% CI)	*I* ^2^ (%)
Clinical							
	LR	6	0.765 (0.613–0.918)	99.2	3	0.801 (0.741–0.861)	92.9
	RF	4			2	0.810 (0.778–0.842)	
	ANN	2	0.798 (0.515–1.081)	98.3	2	0.876 (0.752–1.000)	93.1
	Transformer	1	0.849 (0.821–0.876)				
	RF		0.818 (0.796–0.840)				
	SVM	1	0.777 (0.721–0.834)				
	XGB	4	0.858 (0.814–0.902)	84.8	6	0.833 (0.780–0.886)	90.6
	LightGBM						
	DNN	0	0.746 (0.607–0.885)				
	Cox	3	0.768 (0.669–0.867)	82.1			
	RSF	1	0.794 (0.724–0.865)				
	CatBoost	1	0.841 (0.774–0.907)				
	KNN	1	0.600 (0.565–0.635)				
	DT	2	0.671 (0.565–0.777)	95.2			
	AdaBoost	1	0.671 (0.638–0.704)				
Overall		28	0.777 (0.730–0.825)	97.6	13	0.828 (0.794–0.862)	99.6
Radiomics							
	RF	2	0.786 (0.718–0.855)	53			
	ANN	1	0.763 (0.623–0.903)				
	CNN	1	0.830 (0.730–0.930)				
Overall		4	0.793 (0.752–0.833)	0			
Radiomics + Clinical							
	HNN	1	0.924 (0.847–1.002)				
	LR	4	0.866 (0.751–0.981)	95.4	5	0.868 (0.762–0.974)	93.8
	ANN	1	0.810 (0.770–0.850)		1	0.810 (0.770–0.850)	
	DT	1	0.860 (0.812–0.908)				
	RF	3	0.865 (0.841–0.889)	0	1	0.870 (0.840–0.900)	
	SVM	1	0.790 (0.750–0.830)		2	0.843 (0.735–0.951)	89.3
	DNN				1	0.895 (0.835–0.955)	
Overall		11	0.856 (0.812–0.901)	90.8	10	0.860 (0.809–0.911)	91.5

Abbreviations: AdaBoost, adaptive boosting; ANN, Artificial Neural Network; CatBoost, Categorical Boosting; CI, confidence interval; CNN, Convolutional Neural Network; Cox, Cox proportional hazards model; DNN, Deep Neural Network; DT, Decision Tree; HNN, Hybrid Neural Network; KNN, *K*‐Nearest Neighbors; LASSO, Least Absolute Shrinkage and Selection Operator; LightGBM, Light Gradient Boosting Machine; LR, logistic regression; RF, Random Forest; RSF, Random Survival Forest; SVM, Support Vector Machine; XGB, XGBoost.

#### 3.4.1 Hematoma Expansion

##### 3.4.1.1 Clinical Feature‐Based Models

Six studies developed prediction models for HE using only clinical variables. In the training set (total *n* = 6), the pooled C‐index was 0.751 (95% CI 0.714–0.788), with a summary sensitivity of 0.72 (95% CI 0.67–0.76) and specificity of 0.65 (95% CI 0.59–0.72). The same six studies provided internal or external validation, yielding a pooled C‐index of 0.712 (95% CI 0.674–0.751), sensitivity of 0.69 (95% CI 0.60–0.77), and specificity of 0.66 (95% CI 0.59–0.73).

Funnel plots of the C‐index for prediction models constructed based on clinical features in both the training and validation sets showed no significant publication bias among studies (Figures S1‐S2), with Egger's test significance probabilities of 0.418 and 0.830, respectively.

##### 3.4.1.2 Radiomics‐Only Models

Thirty studies built radiomics‐based models. In the training set, the pooled C‐index reached 0.847 (95% CI 0.818–0.875; *I* ^2^ = 90.7 %), sensitivity of 0.82 (95% CI 0.78–0.85), and specificity of 0.82 (95% CI 0.77–0.86). The validation set (*n* = 30) showed a pooled C‐index of 0.786 (95% CI 0.754–0.819; *I* ^2^ = 78.1 %), sensitivity of 0.76 (95% CI 0.71–0.80), and specificity of 0.79 (95% CI 0.74–0.84).

Funnel plots of the C‐index for prediction models constructed based solely on radiomics features in the training set showed significant publication bias among studies (Figure S3), with an Egger's test significance probability of 0.002. In contrast, funnel plots of the C‐index for radiomics‐only models in the validation set showed no significant publication bias (Figure S4), with an Egger's test significance probability of 0.067.

##### 3.4.1.3 Combined clinical‐radiomics models

Seventeen studies integrated clinical and radiomics features. The training set achieved a pooled C‐index of 0.847 (95% CI 0.808–0.885; *I* ^2^ = 90.5 %), sensitivity of 0.81 (95% CI 0.77–0.85), and specificity of 0.76 (95% CI 0.70–0.82). The validation set (*n* = 23) yielded a pooled C‐index of 0.822 (95% CI 0.789–0.855; *I* ^2^ = 79 %), sensitivity of 0.77 (95% CI 0.72–0.80), and specificity of 0.77 (95% CI 0.73–0.81).

Across all three model categories, logistic regression (LR) was the predominant algorithm; its discriminative performance was non‐inferior to more complex ML techniques (Table 1, Table S2).

Funnel plots of the C‐index for prediction models constructed based on combined clinical‐radiomics features in the training set showed significant publication bias among studies (Figure S5), with an Egger's test significance probability of 0.037. In the validation set, funnel plots of the C‐index for combined clinical‐radiomics models showed no significant publication bias (Figure S6), with an Egger's test significance probability of 0.434.

#### 3.4.2 Poor Functional Outcome

##### 3.4.2.1 Clinical Feature‐Based Models

Twenty‐three studies developed prognostic prediction models for poor functional outcome after ICH using clinical variables alone. In the training set, the pooled C‐index was 0.861 (95% CI 0.824–0.899; *I* ^2^ = 98 %), with a sensitivity of 0.81 (95% CI 0.76–0.86) and a specificity of 0.80 (95% CI 0.73–0.86). Thirty‐one investigations provided internal or external validation, yielding a pooled C‐index of 0.822 (95% CI 0.775–0.868; *I* ^2^ = 97.4 %), sensitivity of 0.74 (95% CI 0.68–0.80), and specificity of 0.84 (95% CI 0.75–0.90).

Funnel plots of the C‐index for prediction models constructed based solely on clinical features in both the training and validation sets showed significant publication bias among studies (Figures S7 and S8), with Egger's test significance probabilities both less than 0.001.

##### 3.4.2.2 Radiomics‐Only Models

Eleven studies constructed radiomics‐based models. In the training set, the pooled C‐index was 0.855 (95% CI 0.811–0.899; *I* ^2^ = 92 %), with a sensitivity of 0.79 (95% CI 0.67–0.87) and a specificity of 0.81 (95% CI 0.76–0.85). The validation set (*n* = 28) showed a summary sensitivity of 0.74 (95% CI 0.68–0.80) and specificity of 0.84 (95% CI 0.75–0.90). A pooled C‐index was not reported for these models in the validation set.

Funnel plots of the C‐index for prediction models constructed based solely on radiomics features in the training set showed no significant publication bias among studies (Figure S9), with an Egger's test significance probability of 0.686. In the validation set, funnel plots of the C‐index for radiomics‐only models showed significant publication bias (Figure S10), with an Egger's test significance probability of 0.002.

##### 3.4.2.3 Combined Clinical‐Radiomics Models

Thirty‐six investigations integrated clinical and radiomic features. The training set achieved a pooled C‐index of 0.856 (95% CI 0.832–0.880; *I* ^2^ = 93.5 %), sensitivity of 0.64 (95% CI 0.46–0.79), and specificity of 0.78 (95% CI 0.69–0.86). The validation set (*n* = 36) yielded a pooled C‐index of 0.850 (95% CI 0.830–0.869; *I* ^2^ = 74.2 %), with insufficient data to summarize sensitivity and specificity.

Across all three model types, LR remained the predominant algorithm; its discriminative performance was not demonstrably inferior to more complex ML techniques (Table 2, Table S3).

Funnel plots of the C‐index for prediction models constructed based on combined clinical‐radiomics features in the training set showed no significant publication bias among studies (Figure S11), with an Egger's test significance probability of 0.932. In the validation set, funnel plots of the C‐index for combined clinical‐radiomics models showed significant publication bias (Figure S12), with an Egger's test significance probability of 0.005.

#### 3.4.3 Mortality

##### 3.4.3.1 Clinical Feature Models

Twenty‐eight studies developed mortality‐prediction models using only clinical variables. In the training set, the pooled C‐index was 0.777 (95% CI 0.730–0.825; *I* ^2^ = 97.6 %), with a summary sensitivity of 0.67 (95% CI 0.59–0.75) and specificity of 0.77 (95% CI 0.71–0.82). Thirteen of these studies provided internal or external validation, yielding a pooled C‐index of 0.828 (95% CI 0.794–0.862; *I* ^2^ = 99.6 %), sensitivity of 0.71 (95% CI 0.60–0.79), and specificity of 0.72 (95% CI 0.60–0.82).

Funnel plots of the C‐index for prediction models constructed based on radiomics features in the training set showed significant publication bias among studies (Figure S13), with an Egger's test significance probability of 0.047. In the validation set, funnel plots of the C‐index for radiomics‐only models showed no significant publication bias (Figure S14), with an Egger's test significance probability of 0.056.

##### 3.4.3.2 Radiomics‐Based Models

Four studies built radiomics‐based models. In the training set, the pooled C‐index was 0.793 (95% CI 0.752–0.833; *I* ^2^ = 0 %), with a sensitivity of 0.87 (95% CI 0.81–0.92) and a specificity of 0.87 (95% CI 0.78–0.92). Since the number of studies in both the training and validation sets was too small, a publication bias analysis was not performed.

##### 3.4.3.3 Combined Clinical‐Radiomics Models

Eleven studies integrated clinical and radiomics features. The training set achieved a pooled C‐index of 0.856 (95% CI 0.812–0.901; *I* ^2^ = 90.8 %), sensitivity of 0.80 (95% CI 0.63–0.90), and specificity of 0.84 (95% CI 0.75–0.90). The validation set (*n* = 10) yielded a pooled C‐index of 0.860 (95% CI 0.809–0.911; *I* ^2^ = 91.5 %), sensitivity of 0.76 (95% CI 0.51–0.91), and specificity of 0.63 (95% CI 0.20–0.92).

Across all three model types, LR remained the predominant algorithm, and its discriminative performance was comparable to that of more complex ML techniques (Table 3, Table S4).

Funnel plots of the C‐index for prediction models constructed based on combined clinical‐radiomics features in the training set showed significant publication bias among studies (Figure S15), with an Egger's test significance probability less than 0.001. In the validation set, funnel plots of the C‐index for combined clinical‐radiomics models showed no significant publication bias (Figure S16), with an Egger's test significance probability of 0.177.

#### 3.4.4 A Review of Other Adverse Outcomes

Beyond the primary outcomes of HE, poor functional outcome, and mortality, this review identified several studies developing models for other clinically relevant endpoints, including functional dependence, neurological deterioration, recurrent ICH (RICH), and in‐hospital mortality. For instance, in predicting functional dependence, Y. Chen, Rivier, et al. (2025) (Yeo et al. 2025) used a Convolutional Neural Network model based on CT radiomics, reporting a C‐index of 0.742 in a validation set. For neurological deterioration, Gao et al. (2022) employed a Random Forest (RF) model with clinical variables, achieving C‐index values of 0.795 and 0.713 in the training and validation sets, respectively. In the prediction of RICH, Luo et al. (2024) applied an LR model combining radiomic and clinical features, which yielded a C‐index of 0.81 in the training set and 0.65 in the validation set. Furthermore, for in‐hospital mortality, (Yeo et al. 2025) used a Decision Tree model integrating radiomics and clinical variables, achieving a C‐index of 0.874 in the training set. Additionally, other studies investigated unfavorable neurological outcomes based on the Glasgow Outcome Scale (GOS), utilizing models such as Artificial Neural Network, XGBoost (XGB), and Least Absolute Shrinkage and Selection Operator (LASSO), with reported C‐index values ranging from 0.701 to 0.921 (Y. Chen, Jiang, et al. 2023; Trevisi et al. 2022; Xing et al. 2023; Zhan et al. 2021). In summary, although these secondary outcomes were studied in smaller cohorts or with varying model performance, they broadened the scope of ML applications in the prognostic prediction of ICH. Future large‐scale prospective studies are warranted to validate their clinical utility and generalizability.

## 4 Discussion

### 4.1 Summary of the Main Findings

Based on 83 externally validated studies (136,840 patients), we identified three dominant outcomes: (1) early HE, where 15 combined clinical‐radiomics models achieved a pooled C‐index of 0.822 (95% CI 0.789–0.855) in the validation set; (2) poor neurological outcome (mRS 3–6 or GOS ≤3), where a meta‐analysis of 18 externally validated models yielded a summary C‐index of 0.850 (95% CI 0.830–0.869) in the validation set; and (3) mortality, where 31 models predicting in‐hospital or 30‐day mortality showed a pooled C‐index of 0.860 (95% CI 0.809–0.911) in the validation set. ML models integrating clinical variables with or without CT radiomics consistently outperformed traditional scores across all domains. In terms of comparative algorithm performance, while LR was the most frequently validated method (*n* = 67) with a maximum pooled C‐index of 0.868, RF and support‐vector machines demonstrated comparable or even higher discriminative ability, with C‐indices of 0.906 and 0.928, respectively. In summary, contemporary ML approaches deliver high discriminative accuracy for the three critical ICH outcomes. It remains unclear, however, to what extent this discriminative performance translates into robust calibration and tangible net clinical benefit, as these critical dimensions were rarely reported in the original studies. Furthermore, these pooled estimates should be interpreted with caution, as the overwhelming majority of original studies were assessed as having a high risk of bias, primarily driven by retrospective designs, limited sample sizes, and insufficient independent validation.

### 4.2 Comparison With Previous Reviews

In the field of ML for predicting adverse outcomes after ICH, several previous reviews have explored related areas in stroke or neuroimaging. For instance, a review by Wang et al. 2025 summarized the applications of radiomics in neuroimaging of stroke, discussing its potential in diagnosis, prognosis prediction, and challenges in standardization (Q. Chen, Xia, et al. 2021). While this review provided a comprehensive overview of radiomics, it did not specifically focus on ML models for ICH adverse outcomes and lacked quantitative synthesis, such as meta‐analysis, limiting the ability to derive pooled estimates of predictive accuracy. Similarly, a review by Verhoeven et al. (2021) on ML in stroke medicine highlighted its role in diagnosis, treatment, and prognosis assessment across stroke types, including ICH (Verhoeven et al. 2021). However, this review was descriptive and did not perform a meta‐analysis for ICH‐specific outcomes, leaving a gap in quantitative evidence.

Regarding poor functional outcomes and mortality in ICH, some reviews have touched on predictive models, but with limitations. For example, a systematic review by Y. Wang et al. (2025). evaluated traditional and ML models for predicting hemorrhagic transformation (HT) after ischemic stroke (Y. Wang, Zhang, Zhang, et al. 2025). Although HT involves hemorrhagic events, the pathophysiology differs from primary ICH, and the review did not address ICH‐specific functional outcomes or mortality. Moreover, while it included ML studies, it was not restricted to ICH and omitted quantitative pooling of data. Another review by Anadani et al. (2021) discussed ML models for predicting outcomes in stroke but covered a broad spectrum without a dedicated meta‐analysis for ICH (Anadani et al. 2021). These reviews often relied on heterogeneous studies with a high risk of bias and did not provide consolidated accuracy metrics for prognostic prediction of ICH.

In contrast, our study addresses these gaps by conducting a systematic review and meta‐analysis exclusively on ML models for predicting adverse outcomes in ICH. We provided quantitative pooled estimates (e.g., AUC or C‐index) for both functional outcomes and mortality, based on a rigorous synthesis of multiple studies. Additionally, we assessed study quality and heterogeneity, offering insights into model generalizability. This approach enhances the evidence base by delivering robust metrics that can inform clinical decisions, such as the superior performance of integrated models combining clinical and radiomic features (Y. Chen, Rivier, et al. 2025). By focusing solely on ICH, our work fills a critical gap and advances the field toward more precise prognostic prediction.

### 4.3 Modeling Variables

In the context of ML models for predicting adverse outcomes in ICH, such as mortality, HE, and functional disability, the choice of modeling variables is critical for model performance, interpretability, and clinical applicability. Based on the synthesized evidence from the included studies, ML approaches primarily utilize three categories of variables: (1) interpretable clinical features, (2) radiomics features derived from medical imaging, and (3) hybrid models combining both clinical and radiomics features. Each category exhibits distinct strengths and limitations, which must be carefully considered to guide future research and clinical implementation.

First, radiomics features, which quantify textural and morphological patterns from NCCT or MRI, have demonstrated high predictive accuracy in numerous studies. For instance, radiomics‐based models outperformed traditional clinical scores in predicting HE and poor functional outcomes (Y. Chen, Rivier, et al. 2025; Huang et al. 2022). However, the implementation of radiomics faces significant challenges that undermine its generalizability. Key issues include the lack of standardization in imaging protocols, such as variations in reconstruction kernels and slice thickness, which can affect feature stability and reproducibility (X. Peng et al. 2022). Additionally, few studies adequately address inter‐observer variability in image segmentation, limiting the consistency of radiomics feature extraction across different settings (Xia et al. 2023). Moreover, radiomics involves high‐dimensional and correlated data, but the robustness of variable selection methods (e.g., filter‐based or embedded techniques) is rarely discussed, leading to potential overfitting and reduced model transportability (M. Wang, Liang, et al. 2024). These factors collectively highlight the need for rigorous validation and standardization before radiomics can be widely adopted.

In contrast, models based on interpretable clinical features, such as age, Glasgow Coma Scale (GCS) score, hematoma volume, and laboratory parameters, offer advantages in transparency and cross‐center consistency. Clinical variables are routinely collected in diverse healthcare settings, facilitating model interpretability and integration into clinical workflows (Bako et al. 2022; Fernando et al. 2021). Importantly, when clinical feature‐based models achieve performance comparable to radiomics‐based models, as observed in some research (Yu et al. 2025), they may be preferable due to their lower complexity and better alignment with clinical reasoning. The growing emphasis on explainable artificial intelligence (XAI) (e.g., SHapley Additive exPlanations [SHAP] analysis) further enhances the value of clinical features by elucidating predictor‐outcome relationships (T. C. Wu et al. 2023). For example, in predicting ICH mortality, clinical variables like GCS and hematoma volume consistently emerged as key predictors across populations (Xia et al. 2022).

Hybrid models that integrate clinical and radiomics features often achieve the highest accuracy, leveraging complementary information (H. L. Wang et al. 2019). However, this approach introduces additional complexity and resource demands. Therefore, in scenarios where clinical features yield non‐inferior performance, prioritizing interpretable clinical models may be more practical for risk assessment tools. Conversely, if radiomics significantly enhances accuracy, as in cases involving subtle imaging biomarkers, the pursuit of high‐performance models is justified (H. Xu et al. 2018). Future studies should focus on standardizing radiomics pipelines, validating models in multicenter cohorts, and incorporating explainability frameworks to bridge the gap between ML innovation and clinical utility.

### 4.4 Influence of Algorithm Selection on Predictive Performance

Our subgroup analyses delineated a clear, albeit complex, relationship between algorithms, predictors, and predictive performance. A pivotal observation was the divergence in model preference between clinical feature‐based and radiomics‐based studies. In models developed solely from clinical features, the predominant approach was LR presented as a nomogram. For predicting poor functional outcome, the pooled validation C‐index for LR was 0.756, which was numerically lower than that of more complex, less interpretable algorithms such as RF (0.883) and XGBoost (0.896). A similar numerical trend was observed for mortality prediction, where XGBoost (0.833) outperformed LR (0.801) in the validation set. This suggests that, for simple, structured clinical data, advanced ensemble or kernel‐based methods can more effectively capture latent nonlinear interactions than a standard linear model.

Conversely, within radiomics‐only and combined clinical‐radiomics models, which inherently handle high‐dimensional data, LR was the algorithm of choice. Despite its relative simplicity, LR demonstrated robust performance across all outcomes, often approaching that of more complex algorithms. For instance, for HE prediction with combined models, the pooled validation C‐index for LR (0.826) was close to that of Support Vector Machine (SVM) (0.891) and XGBoost (0.850). This finding indicates that when the primary modeling challenge is managing a large number of features, regularization methods like LASSO combined with LR can achieve good performance while controlling overfit, offering accuracy that approaches black‐box models.

These findings highlight a critical trade‐off between predictive accuracy and model explainability. While complex models like RF and XGB may offer marginal gains in discriminative power, especially with clinical data, their “black‐box” nature significantly impedes clinical interpretation. LR, regardless of its performance, maintains a crucial advantage in transparency, allowing direct inference of predictor‐outcome relationships via odds ratios or SHAP values. Given that the absolute difference in C‐index between algorithm classes is frequently modest, the pursuit of maximum discrimination should be cautiously weighed against the paramount need for clinical trust and actionable interpretation. Furthermore, the high heterogeneity observed for SVM in radiomics‐only HE prediction (*I* ^2^ = 87.9%) suggests that algorithm performance is highly task‐ and data‐dependent. This reinforces that algorithm type is a qualitative, rather than a quantitative, effect modifier that does not apply to simple hierarchical ranking. Future model development should therefore prioritize methodological rigor, including robust internal and external validation, over algorithm complexity per se, and explore interpretable ML frameworks to make even complex models clinically transparent.

### 4.5 Advantages and Limitations of the Study

This systematic review has several limitations that reflect broader challenges in the field. First, and most importantly, the PROBAST assessment revealed that the vast majority of included studies were at high risk of bias, predominantly originating from participant selection and statistical analysis. Regarding participant selection, most studies employed retrospective or case‐control designs without prospective power calculations, and many were conducted at single centers with limited sample sizes, raising substantial concerns about selection bias, overfitting, and limited generalizability. In the statistical analysis, key issues included insufficient validation, with most studies relying on internal validation rather than independent external validation, and inadequate sample sizes. These inherent methodological limitations introduce a degree of uncertainty that should be carefully weighed when interpreting the pooled estimates. The favorable discriminative performance observed in our meta‐analysis should therefore be viewed as preliminary evidence requiring confirmation in future, rigorously designed prospective studies.

Second, our pooled estimates are heavily driven by internal validation (Lin et al. 2023; Matsumoto et al. 2024), as true external validation in completely independent cohorts was conducted in only a handful of studies. Due to this extreme sparsity, a stratified meta‐analysis by validation type was not feasible, limiting our ability to comprehensively assess model transportability. This methodological gap underscores the imperative for future studies to incorporate rigorous independent external validation.

Third, although formal publication bias assessment using funnel plots and Egger's tests did not reveal statistically significant small‐study effects for the three primary outcomes, some asymmetry was visually observed in certain subgroup analyses with limited numbers of studies. Therefore, the possibility of selective reporting of positive results cannot be entirely ruled out. We also acknowledge the inherent limitations of conventional methods of publication bias assessment in the context of meta‐analyses on prediction models. Funnel plot asymmetry may arise from sources other than publication bias, such as true heterogeneity in model performance due to differences in case mix, outcome definitions, or validation designs, rather than from small‐study effects. Moreover, these tests have low power when the number of included studies is small, as was the case in several of our subgroup analyses. Consequently, the absence of statistically significant asymmetry should not be overinterpreted as definitive evidence of no publication bias.

Fourth, our review was unable to evaluate model calibration or clinical utility, as the vast majority of included studies focused exclusively on discrimination (C‐index/AUC) and did not report calibration metrics (e.g., calibration plots, Hosmer–Lemeshow test, expected‐to‐observed ratios) or decision curve analyses. This reporting gap is a critical limitation of the current literature on ML for the prognostic prediction of ICH, as it precludes assessment of whether predicted probabilities match observed outcomes and whether models yield net clinical benefit across reasonable threshold probabilities. Without such evidence, even models with excellent discrimination may yield systematically biased probability estimates that undermine their reliability for individualized risk stratification in real‐world settings.

Fifth, the clinical interpretability of many ML models remains a barrier. While complex “black‐box” models may offer superior accuracy, the limited application of XAI techniques in the original studies (Pan et al. 2024; S. Y. Peng et al. 2010) hinders clinical trust and understanding of the predictions.

Sixth, the number of studies available for certain meta‐analyses was limited, which may affect the robustness of the pooled estimates. Although our meta‐regression analyses indicated that outcome assessment timepoint was not a statistically significant effect modifier, incomplete reporting of timepoints in some original studies may have reduced the statistical power of these analyses. Additionally, remaining heterogeneity in outcome definitions could not be fully explored through meta‐regression. Moreover, the observed heterogeneity across studies in outcome definitions (e.g., poor functional outcome defined as mRS 3–6 in some studies vs. mRS 4–6 in others; mortality assessed at varying timepoints, such as in hospital, 30 days, 90 days, or 12 months) and in the timing of outcome ascertainment may have influenced the pooled performance estimates, limiting direct comparability between studies. This variability should be carefully considered when interpreting our summary findings. Inconsistent reporting of sensitivity and specificity also limits our ability to conduct a formal bivariate meta‐analysis of these metrics and precludes a quantitative synthesis of feature importance across studies. Future studies should adopt standardized core outcomes for the prognostic prediction of ICH to facilitate the synthesis of more robust evidence.

In conclusion, while this work reveals the promise of ML in the prognostic prediction of ICH, future studies must prioritize larger and multicenter datasets, rigorous external validation, and enhanced model interpretability to advance towards clinically useful tools.

## 5 Conclusions

In conclusion, this study provides consolidated evidence that ML models, particularly those integrating clinical and radiomics features, exhibit promising discriminative ability for predicting key adverse outcomes in patients with spontaneous ICH. However, the evidence base is substantially constrained by the high risk of bias identified in the majority of original studies, predominantly stemming from retrospective or case‐control designs, limited sample sizes, and a pervasive lack of robust independent external validation. These inherent methodological limitations, together with the sparse reporting of calibration metrics and the near absence of clinical utility assessments, attenuate the strength of the currently available evidence. Consequently, the pooled estimates of high discriminative performance presented in this review should be viewed as preliminary and interpreted with appropriate caution. While these models hold potential to inform prognostic stratification and personalized treatment strategies in ICH management, their translation into clinical practice remains contingent upon future research that prioritizes rigorous, prospective, multicenter validation in diverse cohorts, comprehensive reporting of calibration and decision curve analyses, and a steadfast commitment to developing transparent, interpretable, and clinically actionable tools.

## Author Contributions

**Hui Lu**: investigation, data curation, validation, writing – review and editing. **Jianlong Bi**: resources, validation, writing – review and editing. **Weitao Cheng**: conceptualization, data curation, formal analysis, writing – original draft. **Nan Fei**: investigation, data curation, validation, writing – review and editing. **Qi Deng**: conceptualization, data curation, formal analysis, writing – original draft. **Rong He**: software, validation, formal analysis, writing – review and editing. **Yuanyuan Chen**: resources, validation, writing – review and editing. **Wenluo Zhang**: supervision, writing – review and editing.

## Funding

The authors have nothing to report.

## Ethics Statement

The authors have nothing to report.

## Consent

The authors have nothing to report.

## Conflicts of Interest

The authors declare no conflicts of interest.

## Supporting information




**Supplementary File 1**. Literature search strategy
**Table S1**. Characteristics of incorporated studies
**Table S2**. Pooled sensitivity and specificity of machine learning for predicting hematoma expansion
**Table S3**. Pooled sensitivity and specificity of machine learning for predicting poor functional outcomes
**Table S4**. Pooled sensitivity and specificity of machine learning for predicting mortality
**Figure S1** Meta‐analysis funnel plot for clinical feature‐based models for predicting hematoma expansion in the training set
**Figure S2** Meta‐analysis funnel plot for clinical feature‐based models for predicting hematoma expansion in the validation set
**Figure S3** Meta‐analysis funnel plot for radiomics‐based models for predicting hematoma expansion in the training set
**Figure S4** Meta‐analysis funnel plot for radiomics‐based models for predicting hematoma expansion in the validation set
**Figure S5** Meta‐analysis funnel plot for combined clinical‐radiomics models for predicting hematoma expansion in the training set
**Figure S6** Meta‐analysis funnel plot for combined clinical‐radiomics models for predicting hematoma expansion in the validation set
**Figure S7** Meta‐analysis funnel plot for clinical feature‐based models for predicting poor functional outcome in the training set
**Figure S8** Meta‐analysis funnel plot for clinical feature‐based models for predicting poor functional outcome in the validation set
**Figure S9** Meta‐analysis funnel plot for radiomics‐based models for predicting poor functional outcome in the training set
**Figure S10** Meta‐analysis funnel plot for radiomics‐based models for predicting poor functional outcome in the validation set
**Figure S11** Meta‐analysis funnel plot for combined clinical‐radiomics models for predicting poor functional outcome in the training set
**Figure S12** Meta‐analysis funnel plot for combined clinical‐radiomics models for predicting poor functional outcome in the validation set
**Figure S13** Meta‐analysis funnel plot for clinical feature‐based models for predicting mortality in the training set
**Figure S14** Meta‐analysis funnel plot for clinical feature‐based models for predicting mortality in the validation set
**Figure S15** Meta‐analysis funnel plot for combined clinical‐radiomics models for predicting mortality in the training set
**Figure S16** Meta‐analysis funnel plot for combined clinical‐radiomics models for predicting mortality in the validation set

## Data Availability

The original contributions presented in the study are included in the article; further inquiries can be directed to the corresponding author.
