# Supplementary material for: Machine Learning‐Based Prediction of Poor Outcomes in Intracerebral Hemorrhage: A Systematic Review and Meta‐Analysis
Source: Brain Behav. 2026 Jul 31;16(8):e71572. doi: 10.1002/brb3.71572 (PMC13425614; doi:10.1002/brb3.71572)
Supplement: Supplementary file 1 — Literature search strategy Table S1. Characteristics of incorporated studies Table S2. Pooled sensitivity and specificity of machine learning for predicting hematoma expansion Table S3. Pooled sensitivity and specificity of machine learning for predicting poor functional outcomes Table S4. Pooled sensitivity and specificity of machine learning for predicting mortality Figure S1 Meta‐analysis funnel plot for clinical feature‐based models for predicting hematoma expansion in the training set Figure S2 Meta‐analysis funnel plot for clinical feature‐based models for predicting hematoma expansion in the validation set Figure S3 Meta‐analysis funnel plot for radiomics‐based models for predicting hematoma expansion in the training set Figure S4 Meta‐analysis funnel plot for radiomics‐based models for predicting hematoma expansion in the validation set Figure S5 Meta‐analysis funnel plot for combined clinical‐radiomics models for predicting hematoma expansion in the training set Figure S6 Meta‐analysis funnel plot for combined clinical‐radiomics models for predicting hematoma expansion in the validation set Figure S7 Meta‐analysis funnel plot for clinical feature‐based models for predicting poor functional outcome in the training set Figure S8 Meta‐analysis funnel plot for clinical feature‐based models for predicting poor functional outcome in the validation set Figure S9 Meta‐analysis funnel plot for radiomics‐based models for predicting poor functional outcome in the training set Figure S10 Meta‐analysis funnel plot for radiomics‐based models for predicting poor functional outcome in the validation set Figure S11 Meta‐analysis funnel plot for combined clinical‐radiomics models for predicting poor functional outcome in the training set Figure S12 Meta‐analysis funnel plot for combined clinical‐radiomics models for predicting poor functional outcome in the validation set Figure S13 Meta‐analysis funnel plot for clinical feature‐based models for predict [file BRB3-16-e71572-s001.doc]

# Supplementary File 1 Literature search strategy

**1.Pubmed**

| Search number | Query | Results |
| --- | --- | --- |
| #1 | "Cerebral Hemorrhage"[Mesh] | 39,729 |
| #2 | "Machine Learning"[Mesh] | 91,311 |
| #3 | ((((((((((((((((((((((((((((((((((((Cerebral Hemorrhage[Title/Abstract]) OR (Cerebrum Hemorrhage[Title/Abstract])) OR (Cerebrum Hemorrhages[Title/Abstract])) OR (Cerebral Parenchymal Hemorrhage[Title/Abstract])) OR (Cerebral Parenchymal Hemorrhages[Title/Abstract])) OR (Intracerebral Hemorrhage[Title/Abstract])) OR (Intracerebral Hemorrhages[Title/Abstract])) OR (Cerebral Hemorrhages[Title/Abstract])) OR (Cerebral Brain Hemorrhages[Title/Abstract])) OR (brain hemorrhage[Title/Abstract])) OR (brain bleeding[Title/Abstract])) OR (brain haemorrhage[Title/Abstract])) OR (brain microhaemorrhage[Title/Abstract])) OR (brain microhemorrhage[Title/Abstract])) OR (cerebral haemorrhage[Title/Abstract])) OR (cerebral hemorrhage[Title/Abstract])) OR (cerebral microbleed[Title/Abstract])) OR (corpus callosum bleeding[Title/Abstract])) OR (corpus callosum haemorrhage[Title/Abstract])) OR (corpus callosum hemorrhage[Title/Abstract])) OR (encephalorrhagia[Title/Abstract])) OR (hematencephalon[Title/Abstract])) OR (hemorrhagic apoplexy[Title/Abstract])) OR (hemorrhagic stroke[Title/Abstract])) OR (intracerebral bleeding[Title/Abstract])) OR (intracerebral haemorrhage[Title/Abstract])) OR (intracerebral hemorrhage[Title/Abstract])) OR (intracortical haemorrhage[Title/Abstract])) OR (intracortical hemorrhage[Title/Abstract])) OR (intracranial bleeding[Title/Abstract])) OR (intracranial haemorrhage[Title/Abstract])) OR (intracranial haemorrhages[Title/Abstract])) OR (intracranial hemorrhage[Title/Abstract])) OR (intracranial hemorrhages[Title/Abstract])) OR (intraventricular hemorrhage[Title/Abstract])) OR (periventricular haemorrhage[Title/Abstract])) OR (periventricular hemorrhage[Title/Abstract]) | 65,093 |
| #4 | (((((((((((((((((((((((((((((((((((machine learning[Title/Abstract]) OR (artificial intelligence[Title/Abstract])) OR (Transfer Learning[Title/Abstract])) OR (prediction model[Title/Abstract])) OR (Deep learning[Title/Abstract])) OR (ResNet[Title/Abstract])) OR (AlexNet[Title/Abstract])) OR (VGGNet[Title/Abstract])) OR (GoogLeNet[Title/Abstract])) OR (Ensemble Learning[Title/Abstract])) OR (risk model[Title/Abstract])) OR (risk score[Title/Abstract])) OR (random forest[Title/Abstract])) OR (neural network[Title/Abstract])) OR (neural networks[Title/Abstract])) OR (CNN[Title/Abstract])) OR (K-Nearest Neighbor[Title/Abstract])) OR (Support vector machine[Title/Abstract])) OR (SVM[Title/Abstract])) OR (Gradient Boosting Machine[Title/Abstract])) OR (Nomogram[Title/Abstract])) OR (XGBoost[Title/Abstract])) OR (Adaboost[Title/Abstract])) OR (LightGBM[Title/Abstract])) OR (CatBoost[Title/Abstract])) OR (Gradient Boosting[Title/Abstract])) OR (Decision tree[Title/Abstract])) OR (Regression Trees[Title/Abstract])) OR (Naive Bayesian[Title/Abstract])) OR (Multilayer perceptron[Title/Abstract])) OR (Bayesian network[Title/Abstract])) OR (Radiomics[Title/Abstract])) OR (Radiomic[Title/Abstract])) OR (Federated Learning[Title/Abstract])) OR (Federated Learnings[Title/Abstract])) OR (Support Vector Networks[Title/Abstract]) | 466,066 |
| #5 | ("Cerebral Hemorrhage"[Mesh]) OR (((((((((((((((((((((((((((((((((((((Cerebral Hemorrhage[Title/Abstract]) OR (Cerebrum Hemorrhage[Title/Abstract])) OR (Cerebrum Hemorrhages[Title/Abstract])) OR (Cerebral Parenchymal Hemorrhage[Title/Abstract])) OR (Cerebral Parenchymal Hemorrhages[Title/Abstract])) OR (Intracerebral Hemorrhage[Title/Abstract])) OR (Intracerebral Hemorrhages[Title/Abstract])) OR (Cerebral Hemorrhages[Title/Abstract])) OR (Cerebral Brain Hemorrhages[Title/Abstract])) OR (brain hemorrhage[Title/Abstract])) OR (brain bleeding[Title/Abstract])) OR (brain haemorrhage[Title/Abstract])) OR (brain microhaemorrhage[Title/Abstract])) OR (brain microhemorrhage[Title/Abstract])) OR (cerebral haemorrhage[Title/Abstract])) OR (cerebral hemorrhage[Title/Abstract])) OR (cerebral microbleed[Title/Abstract])) OR (corpus callosum bleeding[Title/Abstract])) OR (corpus callosum haemorrhage[Title/Abstract])) OR (corpus callosum hemorrhage[Title/Abstract])) OR (encephalorrhagia[Title/Abstract])) OR (hematencephalon[Title/Abstract])) OR (hemorrhagic apoplexy[Title/Abstract])) OR (hemorrhagic stroke[Title/Abstract])) OR (intracerebral bleeding[Title/Abstract])) OR (intracerebral haemorrhage[Title/Abstract])) OR (intracerebral hemorrhage[Title/Abstract])) OR (intracortical haemorrhage[Title/Abstract])) OR (intracortical hemorrhage[Title/Abstract])) OR (intracranial bleeding[Title/Abstract])) OR (intracranial haemorrhage[Title/Abstract])) OR (intracranial haemorrhages[Title/Abstract])) OR (intracranial hemorrhage[Title/Abstract])) OR (intracranial hemorrhages[Title/Abstract])) OR (intraventricular hemorrhage[Title/Abstract])) OR (periventricular haemorrhage[Title/Abstract])) OR (periventricular hemorrhage[Title/Abstract])) | 81,816 |
| #6 | ("Machine Learning"[Mesh]) OR ((((((((((((((((((((((((((((((((((((machine learning[Title/Abstract]) OR (artificial intelligence[Title/Abstract])) OR (Transfer Learning[Title/Abstract])) OR (prediction model[Title/Abstract])) OR (Deep learning[Title/Abstract])) OR (ResNet[Title/Abstract])) OR (AlexNet[Title/Abstract])) OR (VGGNet[Title/Abstract])) OR (GoogLeNet[Title/Abstract])) OR (Ensemble Learning[Title/Abstract])) OR (risk model[Title/Abstract])) OR (risk score[Title/Abstract])) OR (random forest[Title/Abstract])) OR (neural network[Title/Abstract])) OR (neural networks[Title/Abstract])) OR (CNN[Title/Abstract])) OR (K-Nearest Neighbor[Title/Abstract])) OR (Support vector machine[Title/Abstract])) OR (SVM[Title/Abstract])) OR (Gradient Boosting Machine[Title/Abstract])) OR (Nomogram[Title/Abstract])) OR (XGBoost[Title/Abstract])) OR (Adaboost[Title/Abstract])) OR (LightGBM[Title/Abstract])) OR (CatBoost[Title/Abstract])) OR (Gradient Boosting[Title/Abstract])) OR (Decision tree[Title/Abstract])) OR (Regression Trees[Title/Abstract])) OR (Naive Bayesian[Title/Abstract])) OR (Multilayer perceptron[Title/Abstract])) OR (Bayesian network[Title/Abstract])) OR (Radiomics[Title/Abstract])) OR (Radiomic[Title/Abstract])) OR (Federated Learning[Title/Abstract])) OR (Federated Learnings[Title/Abstract])) OR (Support Vector Networks[Title/Abstract])) | 472,480 |
| #7 | (("Cerebral Hemorrhage"[Mesh]) OR (((((((((((((((((((((((((((((((((((((Cerebral Hemorrhage[Title/Abstract]) OR (Cerebrum Hemorrhage[Title/Abstract])) OR (Cerebrum Hemorrhages[Title/Abstract])) OR (Cerebral Parenchymal Hemorrhage[Title/Abstract])) OR (Cerebral Parenchymal Hemorrhages[Title/Abstract])) OR (Intracerebral Hemorrhage[Title/Abstract])) OR (Intracerebral Hemorrhages[Title/Abstract])) OR (Cerebral Hemorrhages[Title/Abstract])) OR (Cerebral Brain Hemorrhages[Title/Abstract])) OR (brain hemorrhage[Title/Abstract])) OR (brain bleeding[Title/Abstract])) OR (brain haemorrhage[Title/Abstract])) OR (brain microhaemorrhage[Title/Abstract])) OR (brain microhemorrhage[Title/Abstract])) OR (cerebral haemorrhage[Title/Abstract])) OR (cerebral hemorrhage[Title/Abstract])) OR (cerebral microbleed[Title/Abstract])) OR (corpus callosum bleeding[Title/Abstract])) OR (corpus callosum haemorrhage[Title/Abstract])) OR (corpus callosum hemorrhage[Title/Abstract])) OR (encephalorrhagia[Title/Abstract])) OR (hematencephalon[Title/Abstract])) OR (hemorrhagic apoplexy[Title/Abstract])) OR (hemorrhagic stroke[Title/Abstract])) OR (intracerebral bleeding[Title/Abstract])) OR (intracerebral haemorrhage[Title/Abstract])) OR (intracerebral hemorrhage[Title/Abstract])) OR (intracortical haemorrhage[Title/Abstract])) OR (intracortical hemorrhage[Title/Abstract])) OR (intracranial bleeding[Title/Abstract])) OR (intracranial haemorrhage[Title/Abstract])) OR (intracranial haemorrhages[Title/Abstract])) OR (intracranial hemorrhage[Title/Abstract])) OR (intracranial hemorrhages[Title/Abstract])) OR (intraventricular hemorrhage[Title/Abstract])) OR (periventricular haemorrhage[Title/Abstract])) OR (periventricular hemorrhage[Title/Abstract]))) AND (("Machine Learning"[Mesh]) OR ((((((((((((((((((((((((((((((((((((machine learning[Title/Abstract]) OR (artificial intelligence[Title/Abstract])) OR (Transfer Learning[Title/Abstract])) OR (prediction model[Title/Abstract])) OR (Deep learning[Title/Abstract])) OR (ResNet[Title/Abstract])) OR (AlexNet[Title/Abstract])) OR (VGGNet[Title/Abstract])) OR (GoogLeNet[Title/Abstract])) OR (Ensemble Learning[Title/Abstract])) OR (risk model[Title/Abstract])) OR (risk score[Title/Abstract])) OR (random forest[Title/Abstract])) OR (neural network[Title/Abstract])) OR (neural networks[Title/Abstract])) OR (CNN[Title/Abstract])) OR (K-Nearest Neighbor[Title/Abstract])) OR (Support vector machine[Title/Abstract])) OR (SVM[Title/Abstract])) OR (Gradient Boosting Machine[Title/Abstract])) OR (Nomogram[Title/Abstract])) OR (XGBoost[Title/Abstract])) OR (Adaboost[Title/Abstract])) OR (LightGBM[Title/Abstract])) OR (CatBoost[Title/Abstract])) OR (Gradient Boosting[Title/Abstract])) OR (Decision tree[Title/Abstract])) OR (Regression Trees[Title/Abstract])) OR (Naive Bayesian[Title/Abstract])) OR (Multilayer perceptron[Title/Abstract])) OR (Bayesian network[Title/Abstract])) OR (Radiomics[Title/Abstract])) OR (Radiomic[Title/Abstract])) OR (Federated Learning[Title/Abstract])) OR (Federated Learnings[Title/Abstract])) OR (Support Vector Networks[Title/Abstract]))) | 1,365 |

**2.Cochrane**

| Search number | Query | Results |
| --- | --- | --- |
| #1 | MeSH descriptor: [Cerebral Hemorrhage] explode all trees | 1605 |
| #2 | MeSH descriptor: [Machine Learning] explode all trees | 1103 |
| #3 | (Cerebral Hemorrhage):ti,ab,kw OR (Cerebrum Hemorrhage):ti,ab,kw OR (Cerebrum Hemorrhages):ti,ab,kw OR (Cerebral Parenchymal Hemorrhage):ti,ab,kw OR (Cerebral Parenchymal Hemorrhages):ti,ab,kw (Word variations have been searched) | 6240 |
| #4 | (Intracerebral Hemorrhage):ti,ab,kw OR (Intracerebral Hemorrhages):ti,ab,kw OR (Cerebral Hemorrhages):ti,ab,kw OR (Cerebral Brain Hemorrhages):ti,ab,kw OR (brain hemorrhage):ti,ab,kw (Word variations have been searched) | 12456 |
| #5 | (brain bleeding):ti,ab,kw OR (brain haemorrhage):ti,ab,kw OR (brain microhaemorrhage):ti,ab,kw OR (brain microhemorrhage):ti,ab,kw OR (cerebral haemorrhage):ti,ab,kw (Word variations have been searched) | 12776 |
| #6 | (cerebral hemorrhage):ti,ab,kw OR (cerebral microbleed):ti,ab,kw OR (corpus callosum bleeding):ti,ab,kw OR (corpus callosum haemorrhage):ti,ab,kw OR (corpus callosum hemorrhage):ti,ab,kw (Word variations have been searched) | 6300 |
| #7 | (encephalorrhagia):ti,ab,kw OR (hematencephalon):ti,ab,kw OR (hemorrhagic apoplexy):ti,ab,kw OR (hemorrhagic stroke):ti,ab,kw OR (intracerebral bleeding):ti,ab,kw (Word variations have been searched) | 8428 |
| #8 | (intracerebral haemorrhage):ti,ab,kw OR (intracerebral hemorrhage):ti,ab,kw OR (intracortical haemorrhage):ti,ab,kw OR (intracortical hemorrhage):ti,ab,kw OR (intracranial bleeding):ti,ab,kw (Word variations have been searched) | 4808 |
| #9 | (intracranial haemorrhage):ti,ab,kw OR (intracranial haemorrhages):ti,ab,kw OR (intracranial hemorrhage):ti,ab,kw OR (intracranial hemorrhages):ti,ab,kw OR (intraventricular hemorrhage):ti,ab,kw (Word variations have been searched) | 6284 |
| #10 | (periventricular haemorrhage):ti,ab,kw OR (periventricular hemorrhage):ti,ab,kw (Word variations have been searched) | 519 |
| #11 | (machine learning):ti,ab,kw OR (artificial intelligence):ti,ab,kw OR (Transfer Learning):ti,ab,kw OR (prediction model):ti,ab,kw OR (Deep learning):ti,ab,kw (Word variations have been searched) | 37773 |
| #12 | (ResNet):ti,ab,kw OR (AlexNet):ti,ab,kw OR (VGGNet):ti,ab,kw OR (GoogLeNet):ti,ab,kw OR (Ensemble Learning):ti,ab,kw (Word variations have been searched) | 267 |
| #13 | (risk model):ti,ab,kw OR (risk score):ti,ab,kw OR (random forest):ti,ab,kw OR (neural network):ti,ab,kw OR (neural networks):ti,ab,kw (Word variations have been searched) | 107781 |
| #14 | (CNN):ti,ab,kw OR (K-Nearest Neighbor):ti,ab,kw OR (Support vector machine):ti,ab,kw OR (SVM):ti,ab,kw OR (Gradient Boosting Machine):ti,ab,kw (Word variations have been searched) | 2280 |
| #15 | (Nomogram):ti,ab,kw OR (XGBoost):ti,ab,kw OR (Adaboost):ti,ab,kw OR (LightGBM):ti,ab,kw OR (CatBoost):ti,ab,kw (Word variations have been searched) | 2166 |
| #16 | (Gradient Boosting):ti,ab,kw OR (Decision tree):ti,ab,kw OR (Regression Trees):ti,ab,kw OR (Naive Bayesian):ti,ab,kw OR (Multilayer perceptron):ti,ab,kw (Word variations have been searched) | 1983 |
| #17 | (Bayesian network):ti,ab,kw OR (Radiomics):ti,ab,kw OR (Radiomic):ti,ab,kw OR (Federated Learning):ti,ab,kw OR (Federated Learnings):ti,ab,kw (Word variations have been searched) | 1462 |
| #18 | (Support Vector Networks):ti,ab,kw (Word variations have been searched) | 160 |
| #19 | #1 or #3 or #4 or #5 or #6 or #7 or #8 or #9 or #10 | 17896 |
| #20 | #2 or #11 or #12 or #13 or #14 or #15 or #16 or #17 or #18 | 134409 |
| #21 | #19 and #20 | 2878 |

**3.Embase**

| Search number | Query | Results |
| --- | --- | --- |
| #2 | 'brain hemorrhage'/exp | 205048 |
| #3 | 'machine learning'/exp | 577685 |
| #4 | machine AND learning OR 'artificial intelligence':ab,ti OR 'transfer learning':ab,ti OR 'prediction model':ab,ti OR 'deep learning':ab,ti OR resnet:ab,ti OR alexnet:ab,ti OR vggnet:ab,ti OR googlenet:ab,ti OR 'ensemble learning':ab,ti OR 'risk model':ab,ti OR 'risk score':ab,ti OR 'random forest':ab,ti OR 'neural network':ab,ti OR 'neural networks':ab,ti OR cnn:ab,ti OR 'k-nearest neighbor':ab,ti OR 'support vector machine':ab,ti OR svm:ab,ti OR 'gradient boosting machine':ab,ti OR nomogram:ab,ti OR xgboost:ab,ti OR adaboost:ab,ti OR lightgbm:ab,ti OR catboost:ab,ti OR 'gradient boosting':ab,ti OR 'decision tree':ab,ti OR 'regression trees':ab,ti OR 'naive bayesian':ab,ti OR 'multilayer perceptron':ab,ti OR 'bayesian network':ab,ti OR radiomics:ab,ti OR radiomic:ab,ti OR 'federated learning':ab,ti OR 'federated learnings':ab,ti OR 'support vector networks':ab,ti | 581599 |
| #5 | 'brain hemorrhage' OR 'cerebrum hemorrhage':ab,ti OR 'cerebrum hemorrhages':ab,ti OR 'cerebral parenchymal hemorrhage':ab,ti OR 'cerebral parenchymal hemorrhages':ab,ti OR 'intracerebral hemorrhages':ab,ti OR 'cerebral hemorrhages':ab,ti OR 'cerebral brain hemorrhages':ab,ti OR 'brain bleeding':ab,ti OR 'brain haemorrhage':ab,ti OR 'brain microhemorrhage':ab,ti OR 'cerebral haemorrhage':ab,ti OR 'cerebral hemorrhage':ab,ti OR 'cerebral microbleed':ab,ti OR 'corpus callosum haemorrhage':ab,ti OR 'corpus callosum hemorrhage':ab,ti OR hematencephalon:ab,ti OR 'hemorrhagic apoplexy':ab,ti OR 'hemorrhagic stroke':ab,ti OR 'intracerebral bleeding':ab,ti OR 'intracerebral haemorrhage':ab,ti OR 'intracerebral hemorrhage':ab,ti OR 'intracortical hemorrhage':ab,ti OR 'intracranial bleeding':ab,ti OR 'intracranial haemorrhage':ab,ti OR 'intracranial haemorrhages':ab,ti OR 'intracranial hemorrhage':ab,ti OR 'intracranial hemorrhages':ab,ti OR 'brain hemorrhage':ab,ti OR 'periventricular haemorrhage':ab,ti OR 'periventricular hemorrhage':ab,ti | 168668 |
| #6 | #2 OR #5 | 216805 |
| #7 | #3 OR #4 | 822483 |
| #8 | #6 AND #7 | 3873 |

**4.Web of science**

| Search number | Query | Results |
| --- | --- | --- |
| #1 | TS=(Cerebral Hemorrhage OR Cerebrum Hemorrhage OR Cerebrum Hemorrhages OR Cerebral Parenchymal Hemorrhage OR Cerebral Parenchymal Hemorrhages OR Intracerebral Hemorrhage OR Intracerebral Hemorrhages OR Cerebral Hemorrhages OR Cerebral Brain Hemorrhages OR brain hemorrhage OR brain bleeding OR brain haemorrhage OR brain microhaemorrhage OR brain microhemorrhage OR cerebral haemorrhage OR cerebral hemorrhage OR cerebral microbleed OR corpus callosum bleeding OR corpus callosum haemorrhage OR corpus callosum hemorrhage OR encephalorrhagia OR hematencephalon OR hemorrhagic apoplexy OR hemorrhagic stroke OR intracerebral bleeding OR intracerebral haemorrhage OR intracerebral hemorrhage OR intracortical haemorrhage OR intracortical hemorrhage OR intracranial bleeding OR intracranial haemorrhage OR intracranial haemorrhages OR intracranial hemorrhage OR intracranial hemorrhages OR intraventricular hemorrhage OR periventricular haemorrhage OR periventricular hemorrhage ) | 124836 |
| #2 | TS=(machine learning OR artificial intelligence OR Transfer Learning OR prediction model OR Deep learning OR ResNet OR AlexNet OR VGGNet OR GoogLeNet OR Ensemble Learning OR risk model OR risk score OR random forest OR neural network OR neural networks OR CNN OR K-Nearest Neighbor OR Support vector machine OR SVM OR Gradient Boosting Machine OR Nomogram OR XGBoost OR Adaboost OR LightGBM OR CatBoost OR Gradient Boosting OR Decision tree OR Regression Trees OR Naive Bayesian OR Multilayer perceptron OR Bayesian network OR Radiomics OR Radiomic OR Federated Learning OR Federated Learnings OR Support Vector Networks ) | 4092503 |
| #3 | #1 AND #2 | 16981 |

**Table S1. Characteristics of incorporated studies**

| **No.** | **DOI** | **Author** | **Year** | **Country** | **Research Type** | **Patient Source** | **Type of Cerebral Hemorrhage** | **Treatment** | Objective Event | Follow-up(d) | Image | events | samplesize | Validation Set Generation Method | Variable Selection Method | Type of Model Used | Modeling Variable Combination |
| --- | --- | --- | --- | --- | --- | --- | --- | --- | --- | --- | --- | --- | --- | --- | --- | --- | --- |
| 1 | 10.1007/s00330-021-07828-7 | Zuhua Song | 2021 | China | Cohort study | Multicenter | spontaneous intracerebral hemorrhage (sICH) | not mentioned | Poor prognosis (mRS 4-6) | 90 | CT | 200 | 435 | Internal verification+external verification | Minimum Redundancy Maximum Relevance (mRMR); LASSO (Least Absolute Shrinkage and Selection Operator); Single factor analysis; Multi factor logistic regression | Logistic regression model; Radiomics score (Rad score, linear combination calculated based on LASSO regression coefficients) | Clinical features (midline shift, onset to NCCT time, GCS score, serum glucose, uric acid); Radiomics features (12 screened radiomics features, including histogram features, texture features, GLCM, RLM, morphological factors, etc.) |
| 2 | 10.1016/j.ejrad.2024.111871 | Xiaona Xia | 2025 | China | Case-control | Multicenter | spontaneous intracerebral hemorrhage (sICH) | not mentioned | Poor prognosis (mRS 4-6) | 90 | CT | 164 | 506 | external validation | LASSO algorithm; Spearman correlation analysis (r ≥ 0.90 to exclude redundant features); Intra group correlation coefficient (ICC<0.75 excluding unreliable features); Single factor and stepwise multiple factor logistic regression analysis (clinical feature screening) | Logistic regression (based on L2 regularization); Radiomics scoring models (RM, 6 models based on different VOI); Clinical Semantic Model (CSM); Clinical Semantics Radiomics Model (CSRM); Nomogram | Clinical features (age, GCS score IVH、PHE、MLS）； Radiomics features (15, including texture features, shape features, first-order features, from within and around the hematoma); Clinical features+radiomics features |
| 3 | 10.3389/fneur.2022.974183 | Wei Xu | 2022 | China | Case-control | Single center | spontaneous intracerebral hemorrhage (sICH) | not mentioned | Hematoma enlargement | 1 | CT | 103 | 388 | external validation | LASSO algorithm (radiomics features); Single factor analysis combined with stepwise logistic regression (clinical and radiological characteristics) | Logistic regression; Radiomics scoring model (R-score); Nomogram | Clinical features (baseline GCS score NLR、 The time from onset to first NCCT; Radiological features (mixed sign, low-density sign, midline shift); Radiomics features (3, including Original_GLRLM_GrayLevelNonUniformity、Wavelet-LLL_GLRLM_ShortRunEmphasis、Wavelet-LLL_NGTDM_Contrast）； Clinical, radiological, and radiomic features |
| 4 | 10.1016/j.wneu.2021.10.129 | ZhiMing Zhou | 2022 | China | Cohort study | Multicenter | spontaneous intracerebral hemorrhage (sICH) | not mentioned | Poor prognosis (mRS 4-6) | 30 | CT | 136 | 326 | Internal verification+external verification | Univariate analysis (independent sample t-test or Mann Whitney U test); LASSO（least absolute shrinkage and selection operator） | Multivariate logistic regression | Clinical features (age, onset to CT time, Glasgow Coma Scale, midline shift); Radiomics (R-score composed of 4 radiomics features); Radiomics+clinical features |
| 5 | 10.1016/j.wneu.2023.11.002 | Lei Pei | 2024 | China | Cohort study | Single center | spontaneous intracerebral hemorrhage (sICH) | not mentioned | Poor prognosis (mRS 3-6) | not mentioned | CT | 216 | 483 | internal validation | LASSO regression (radiomics features); Univariate analysis+stepwise multivariate logistic regression (clinical features) | Logistic Regression (LR, Radiomics Model); Logistic regression (clinical model and combined model) | Clinical features (4 items: gender, IVH, GCS score, ICH volume); Radiomics (8 items: 2 shape features, 1 GLDM feature, 1 GLCM feature, 3 GLSZM features, 1 GLRLM feature); Radiomics+Clinical Features (Combined Model) |
| 6 | 10.1016/j.wneu.2023.12.160 | Zhixian Luo | 2024 | China | Cohort study | Single center | spontaneous intracerebral hemorrhage (sICH) | not mentioned | Recurrent cerebral hemorrhage (RICH) | 1826 | CT | not mentioned | not mentioned | internal validation | Minimum Redundancy Maximum Relevance（MRMR）; LASSO（Least Absolute Shrinkage and Selection Operator）; Univariate analysis (chi square test, Student's t-test, Wilcoxon rank sum test); Multivariate logistic regression | Logistic regression (LR); Support Vector Machine (SVM); Nomogram | Clinical features (platelet count, AST level, bleeding site); Radiomics features (14, including texture features such as GLCM, FIRSTORDER, GLRLM, NGTDM, GLSZM, etc.); Radiomics+clinical features (Rad score+platelet count+AST level+bleeding site) |
| 7 | 10.1038/s41598-024-69249-y | Xuelin Song | 2024 | China | Case-control | Single center | spontaneous intracerebral hemorrhage (sICH) | conservative treatment | Poor prognosis (mRS 3-6) | 90 | CT |  | 996 | internal validation | Mann Whitney U test; LASSO | Logistic regression; Random Forest (for stability verification) | Clinical features (combined intraventricular hemorrhage, gender, baseline ICH volume, CT time AST/ALT）; Radiomics (radiomics features of hematoma and surrounding tissues 5mm, 10mm, 15mm); Radiomics+Clinical Features (Clinical Radiomics Column Chart) |
| 8 | 10.1177/08465371231168383 | Zejia Frank Chen | 2023 | Canada; Italy; USA | Cohort study | Multicenter | spontaneous intracerebral hemorrhage (sICH) | conservative treatment | Hematoma enlargement (volume exceeding baseline by 33% or 6mL) | 1 | CT | 452 | 1268 | internal validation | Single factor logistic regression analysis; Method based on P-value sorting and exclusion of relevant features (conducted in RStudio) | Multiple logistic regression model | Clinical characteristics (age, gender, time from onset to CT, hypertension, diabetes, kidney disease, warfarin use, GCS score); <br>NCCT radiological features (Barras density and irregularity, island sign, black hole sign, mixing sign, liquid level, any low density, vortex sign); <br>Radiomics features (1070 features, 5 were ultimately selected) |
| 9 | 10.1016/j.acra.2020.02.021 | Qian Chen | 2020 | China | Cohort study | Single center | spontaneous intracerebral hemorrhage (sICH) | not mentioned | Hematoma enlargement (volume exceeding baseline by 33% or 6mL) | 3 | CT | 708 | 3459 | internal validation | LASSO algorithm; Stepwise logistic regression analysis | Logistic regression model; Radiomics score (R-score) | Clinical characteristics (gender, GCS score, baseline NCCT time); Radiomics features (Frequency Size, HaralickCorrelation All Direction_offset1_SD, SizeZone Variability); Clinical features+radiomics features |
| 10 | 10.1007/s00330-024-10921-2 | Fei Yu | 2025 | China | Case-control | Multicenter | spontaneous intracerebral hemorrhage (sICH) | not mentioned | Hematoma enlargement | 1 | CT | 756 | 2613 | Internal verification+external verification | T-test; LASSO regression; Pearson correlation analysis | Logistic Regression（LR）； k-Nearest Neighbors（KNN）； Support Vector Machines（SVM）； Decision Trees（DT）； Random Forests（RF）； Linear Discriminant Analysis（LDA）； Quadratic Discriminant Analysis（QDA）； Naive Bayes（NB）， Finally, LR was chosen to construct a radiomics model | Clinical features (admission GCS score, smoking history, etc.); Radiomics features (29); Radiomics+Clinical Features (Mixed Model) |
| 11 | 10.21037/qims-22-128 | Yuanliang Xie | 2022 | China | Cohort study | Multicenter | spontaneous intracerebral hemorrhage (sICH) | Conservative treatment or surgery | Poor prognosis (mRS 4-6) | 30 | CT | 255 | 690 | Internal verification+external verification | LASSO algorithm (radiomics features); Single factor logistic regression+stepwise logistic regression (clinical characteristics and mixed model) | Logistic regression (clinical model, radiomics model, mixed model) | Clinical features (hematoma expansion, intraventricular hemorrhage, hematoma location, etc.); Radiomics features (9 quantitative features, including morphology, texture, etc.); Radiomics+Clinical Features (Mixed Model) |
| 12 | 10.1016/j.ejro.2024.100618 | Antonio López-Rueda | 2024 | Spain | Cohort study | Single center | spontaneous intracerebral hemorrhage (sICH) | not mentioned | mortality rate | not mentioned | CT | 105 | 315 | internal validation | DropOut (Pearson correlation coefficient>0.6 excluded); SelectKBest (ANOVA F-value); L1 (Lasso regularization); L2 (Ridge regularization) | Random Forest（RF）； K-Nearest Neighbors（KNN）； Support Vector Machines（SVM） | Radiomics features (105, including 19 intensity, 10 shape, and 76 texture) |
| 13 | 10.1016/j.jstrokecerebrovasdis.2024.107979 | Menghui Wang | 2024 | China | Cohort study | Multicenter | spontaneous intracerebral hemorrhage (sICH) | not mentioned | Hematoma enlargement | 3 | CT | 101 | 490 | Internal verification+external verification | Student's t-test; Mann Whitney U test; LASSO； mRMR | NnU Net (segmentation model); Multiple logistic regression (predictive model) | Clinical features (age, gender, GCS score, baseline NCCT time, blood pressure, smoking, alcohol consumption, comorbidities, anticoagulant/antiplatelet use, etc.) NLR、 14 items including serum calcium, IVH, etc; Radiomics features (including+2mm peri hematoma radiomics features within the hematoma, a total of 1210 items were screened); Radiomics+clinical features |
| 14 | 10.3389/fnins.2021.766228 | Zhiming Zhou | 2021 | China | Cohort study | Multicenter | spontaneous intracerebral hemorrhage (sICH) | Conservative treatment or surgery | Poor prognosis (mRS 3-6) | 180 | CT | 413 | 886 | external validation | Univariate analysis; LASSO Regression (Least Absolute Shrinkage and Selection Operator Regression) | Multivariate logistic regression; Radiomics score (Rad score, calculated based on LASSO regression coefficients) | Clinical characteristics (Glasgow Coma Scale score, hematoma enlargement, hematoma location, hematoma volume, diabetes, onset to CT time, white blood cell count, serum glucose, midline shift); Radiomics (10 optimal radiomics features); Radiomics+Clinical Features (Combination Model) |
| 15 | 10.3348/kjr.2020.0254 | Zuhua Song | 2021 | China | Case-control | Single center | spontaneous intracerebral hemorrhage (sICH) | not mentioned | Hematoma enlargement | 1 | CT | 440 | 1044 | internal validation | Mann Whitney U test; Spearman correlation analysis; LASSO； Stepwise Regression Based on Akaike Information Criterion | LR (logistic regression); NB (na ï ve Bayes); RF (random forest); SVM (Support Vector Machine); KNN (k-nearest neighbors algorithm) | Clinical characteristics (4 items: gender, diabetes, platelet count, apoA-I); Radiomics (9 items: 3 RLM features, 2 GLSZM features, 2 GLCM features, 1 morphological feature, 1 histogram feature); Radiomics+clinical features+imaging signs (4 imaging signs: mixed sign, black hole sign, vortex sign, uneven density) |
| 16 | 10.1007/s00062-021-01062-w | Hui Li | 2022 | China | Case-control | Single center | spontaneous intracerebral hemorrhage (sICH) | not mentioned | Hematoma enlargement (volume exceeding baseline by 33% or 6mL) | 3 | CT | not mentioned | 774 | internal validation | mRMR; LASSO | Logistic regression (constructing radiomics models, clinical models, radiomics column charts) | Clinical characteristics (gender, age, initial GCS score, etc.); Radiomics (12 radiomics features); Radiomics+clinical features |
| 17 | 10.1016/j.diii.2023.04.008 | Xiaoyu Huang | 2023 | China | Cohort study | Multicenter | spontaneous intracerebral hemorrhage (sICH) | not mentioned | Poor prognosis (mRS 4-6) | 90 | CT | 2109 | 3294 | Internal verification+external verification | Wilcoxon rank sum test (univariate); Multivariate logistic regression analysis (multiple factors) | Logistic Regression Model (LR) | Clinical features (age, GCS score, blood glucose level, bleeding site); Radiomics (7 radiomics features); Radiomics+clinical features |
| 18 | 10.1117/12.2611847 | Seymour SE | 2022 | US | Case-control | Single center | spontaneous intracerebral hemorrhage (sICH) | not mentioned | Hematoma enlargement | not mentioned | CT | 280 | 800 | internal validation | lasso regression； customized selection（for NCCT: 4 shape-based features specified by hypothesis；for MRI: lasso regression） | Support Vector Machine (SVM)； Naïve Bayes (NB)； Decision Tree (DT)； Random Forest (RF)； Logistic Regression (LR)； K-Nearest Neighbor (KNN)； Multilayer Perceptron (MLP) | Radiomics (NCCT: 4 shape based features: lean and major axis length, elongation, flatness; MRI: 3 shape based features, 1 first order statistics feature, and 2 gray level coolness matrix features) |
| 19 | 10.1007/s10072-022-06528-4 | Te‑Chang Wu | 2023 | China Taiwan; USA | Case-control | Single center | spontaneous intracerebral hemorrhage (sICH) | not mentioned | Poor prognosis (mRS 4-6) | 0 | CT | 240 | 498 | internal validation | SVM (Support Vector Machine) combined with Radial Basis Function Kernel | Support Vector Machine (SVM) is used for radiomics models; Multivariate logistic regression for clinical models and combination models | Clinical characteristics (age, gender, onset time of symptoms, hypertension, diabetes, smoking, drinking, use of antiplatelet/anticoagulant drugs, bleeding tendency, GCS, systolic/diastolic blood pressure in emergency, etc.); Radiomics (radiomics features extracted from IPH and IPH+IVH); Radiomics+clinical features |
| 20 | 10.3389/fneur.2023.1053846 | Jing Wang | 2023 | China | Cohort study | Multicenter | spontaneous intracerebral hemorrhage (sICH) | not mentioned | Poor prognosis (mRS 4-6) | 30 | CT | 473 | 1168 | Internal verification+external verification | Student’s t-test； LASSO； 10-fold cross-validation； Univariate analysis (P<0.1 included in multivariate analysis); Multivariate logistic regression analysis (P<0.05 identified as independent risk factor) | Logistic regression model (enter method); Radiomics scoring (Rad score, linear model constructed based on 6 optimal radiomics features) | Clinical features (GCS score, age, serum glucose, creatinine, baseline HICH volume, midline shift, black hole sign, whether or not surgical intervention was selected as independent risk factors through univariate and multivariate analysis, and included in the baseline HICH volume to construct a clinical model); <br>Radiomics (automatically extracting 396 radiomics features from VOI, and screening 6 optimal features through ICC stability evaluation, Student's t-test, LASSO, and 10 fold cross validation): cluster prominence feature、cluster shade feature、MinIntensity feature、correlation feature、volumeCC feature、low-intensity large area emphasis feature， Building Rad core; <br>Radiomics+clinical features (combined with GCS score, baseline HICH volume, midline shift, black hole sign, Rad score to construct a joint model) |
| 21 | 10.1002/brb3.2085 | Xinghua Xu | 2021 | China | Cohort study | Single center | spontaneous intracerebral hemorrhage (sICH) | not mentioned | Poor prognosis (mRS 3-6) | 180 | CT |  | 1620 | internal validation | Variance threshold; SelectKBest； LASSO regression; Principal Component Analysis (PCA); Covariance analysis; cluster analysis | Support Vector Machine (SVM); K-Nearest Neighbor (KNN); Logistic Regression (LR); Decision Tree (DT); Random Forest (RF); XGBoost | Radiomics (18 radiomics features, including 4 first-order statistical features, 4 shape features, and 10 texture features) |
| 22 | 10.1007/s00330-021-07826-9 | Stefan Pszczolkowski | 2021 | UK; Malaysia | Cohort study | Multicenter | spontaneous intracerebral hemorrhage (sICH) | conservative treatment | Poor prognosis (mRS 3-6) | 90 | CT | 790 | 2076 | internal validation | Correlation filtering (removing variables with high average absolute correlation when absolute correlation>0.9); Elastic network regularization (alpha parameter grid search) | Generalized Linear Model (GLM), Elastic Network Regularization (Ridge/LASSO Hybrid, H2O Platform v3.26.0.2) | ① Radiomics features: 754 items (218 items retained after ComBat coordination and correlation screening)<br>② Clinical features: age, gender, symptom to scan time, baseline hematoma volume, antiplatelet use, ultra early hematoma growth rate<br>③ Radiomics+clinical feature combination<br> |
| 23 | 10.3389/fnins.2023.1225342 | Stefan P. Haider | 2023 | USA; Germany; UK | Cohort study | Multicenter | spontaneous intracerebral hemorrhage (sICH) | not mentioned | Hematoma enlargement (volume exceeding baseline by 33% or 6mL) | 90 | CT | 488 | 1794 | external validation | LASSO (Least Absolute Shrinkage and Selection Operator Regularized Logistic Regression) | Logistic Regression, LASSO-LR） | Radiomics features (1130, including shape, first-order, and texture features); Clinical variables (5, gender, baseline NIHSS score, GCS score, platelet count, blood glucose level); Visual markers (8); BAT rating |
| 24 | 10.1259/bjr.20201047 | CHENYI ZHAN | 2021 | China | Cohort study | Single center | spontaneous intracerebral hemorrhage (sICH) | not mentioned | Neurological dysfunction (GOS 1-3) | not mentioned | CT | 187 | 564 | internal validation | LASSO； Single factor analysis; multivariate analysis | Logistic regression (LASSO logistic regression); Radiomics scoring (R-score, Rad score) | Clinical characteristics (age, gender, hypertension history, diabetes history, ischemic stroke history, cerebral hemorrhage history, GCS score, time from symptom onset to CT, hematoma location, whether there is IVH, hematoma volume, black hole sign, mixed sign, island sign, satellite sign); Radiomics (396 features, ultimately selecting 3 features: kurtosis、HaralickCorrelation_AllDirection_offset1_SD、ShortRunHighGreyLevelEmphasis_AllDirection_offset4_SD）； Radiomics+clinical features |
| 25 | 10.21037/atm-21-6158 | Xia X | 2022 | China | Case-control | Multicenter | spontaneous intracerebral hemorrhage (sICH) | not mentioned | Hematoma enlargement | 2 | CT | 432 | 1868 | Internal verification+external verification | ICC (intra group correlation coefficient, retaining ICC ≥ 0.8); ANOVA (one-way analysis of variance, P<0.05）； LASSO (Least Absolute Shrinkage and Selection Operator) | Logistic regression (LR); Comparative models include Na ï ve Bayes（NB）、Random Forest（RF）、Support Vector Machine（SVM）、Gradient Boosting Decision Tree（GBDT） | Clinical features (age, GCS score, onset to baseline CT time, serum glucose, D-dimer, baseline hematoma volume, previous anticoagulant/antiplatelet therapy, etc.); Radiomics features (first-order features, shape features, texture features, a total of 20); Clinical features+radiomics features (CSRM model) |
| 26 | 10.1016/j.nicl.2022.103242 | Xiaoyu Huang | 2022 | China | Cohort study | Multicenter | spontaneous intracerebral hemorrhage (sICH) | not mentioned | Poor prognosis (mRS 4-6) | 90 | CT | 703 | 1098 | Internal verification+external verification | Wilcoxon rank sum test (p<0.01); Multivariate logistic regression (p<0.05) | Logistic Regression (LR); Radiomics score (Rad score, calculated based on multiple logistic regression coefficients) | Clinical features (age, bleeding volume, bleeding location, GCS score, etc.); Radiomics features (214 features of hematoma and periatomatal edema area, ultimately selected as 12); Radiomics+clinical features |
| 27 | 10.3390/diagnostics12112755 | Te-Chang Wu | 2022 | China Taiwan; USA | Case-control | Single center | spontaneous intracerebral hemorrhage (sICH) | not mentioned | Hematoma enlargement | 1 | CT | 115 | 254 | internal validation | SVM (Gaussian Radial Basis Function Kernel); ICC>0.8 | Support Vector Machine (SVM) | Radiomics (IPH radiomics features 5; 6 radiomics features of IPH+IVH |
| 28 | 10.3389/fnins.2024.1394795 | Zhiming Zhou | 2024 | China | Cohort study | Multicenter | spontaneous intracerebral hemorrhage (sICH) | not mentioned | Hematoma enlargement | 1 | CT | 531 | 1470 | external validation | Single factor analysis; Elastic network regression | Elastic web regression (used for feature selection and constructing Radscore); Logistic regression (used to construct the composite model Radscore-HEA-PHE) | Radiomics (radiomics features of hematoma and PHE); Clinical features (ICH onset time, GCS score, baseline hematoma volume, hematoma shape, hematoma density, midline shift) |
| 29 | 10.1101/2024.05.22.24307743 | Liang J.J. | 2024 | China | Cohort study | Multicenter | spontaneous intracerebral hemorrhage (sICH) | Conservative treatment or surgery | Poor prognosis (mRS 3-6) | 365 | CT | not mentioned | not mentioned | external validation | Spearman correlation+L1 regularization (LASSO) | Logistic Regression (scikit learn) | Clinical features (age, gender, hypertension, GCS, etc.); Radiomics features (1454); Image location features (192 brain regions) |
| 30 | 10.1007/s00330-019-06378-3 | Xie HH | 2019 | China | Cohort study | Single center | spontaneous intracerebral hemorrhage (sICH) | not mentioned | Hematoma enlargement | 1 | CT | 324 | 753 | internal validation | LASSO | Logistic regression (including radiomics score) | Radiomics: 12-22 LASSO screening features; Radiology: NCCT signs such as volume, morphology, density, spin/blend/island, etc; Union: Radiological variables+rad score |
| 1 | 10.1016/j.acra.2024.05.035 | Haoyi Ye | 2024 | China | Cohort study | Single center | spontaneous intracerebral hemorrhage (sICH) | conservative treatment | Hematoma enlargement (volume exceeding baseline by 33% or 6mL) | 1 | CT | 590 | 2030 | internal validation | Univariate analysis (clinical features); LASSO regression (radiomics feature screening); Multivariate logistic regression (integrating clinical and radiomic features) | Logistic Regression (LR), Random Forest (RF), Support Vector Machine (SVM), Extreme Gradient Boosting (XGBoost) | Clinical features: Initial GCS score, history of warfarin use<br>Radiomics features: 6 (Maximum2DDiameterSlice, SurfaceVolumeRRatio, RunLength Non Uniformity, LargeAreaEmphasis, LargeAreaHighGrayLevelEmphasis, SmallAreaLowGrayLevelEmphasis) |
| 2 | 10.1186/s12911-023-02293-2 | Yuxin Wang | 2023 | China | Cohort study | Single center | spontaneous intracerebral hemorrhage (sICH) | Conservative treatment is the main approach | mortality rate | 7 | Non-image data | 3848 | 11960 | internal validation | LASSO regression (preliminary screening of 19 variables); <br>Five fold cross validation combined with AIC criterion (Cox model); <br>Minimum Depth Method (RSF Model) | Random Survival Forest (RSF); Cox proportional hazards regression model | Clinical characteristics (19 in total): marital status, peripheral vascular disease (PVD), severe liver disease, uncomplicated diabetes, age, heart rate, mean blood pressure (MBP), body temperature, respiratory rate (RR), platelet count, white blood cell count (WBC), anion gap, creatinine, blood sugar, blood sodium, blood potassium, prothrombin time (PT), Glasgow coma score (GCS), weight<br>(16 RSF models and 18 Cox models were finally included, see Table 1 of the literature and the results for details) |
| 3 | 10.1097/JS9.0000000000000852 | Kaiwen Wang | 2023 | China | Cohort study | Multicenter | spontaneous intracerebral hemorrhage (sICH) | Conservative treatment or surgery | Poor prognosis (mRS 4-6) | 180 | CT | 128 | 794 | internal validation | Single factor logistic regression; Multi factor logistic regression (backward method); Decision Tree Algorithm (rpart Package, Gini Impurity Criterion) | Decision tree (DT, built in R software based on the rpart package) | Clinical features: History of ischemic cardiovascular and cerebrovascular disease (ICCD), renal dysfunction, dual antiplatelet therapy (DAPT), admission GCS score, hematoma volume<br>(all independent risk factors screened by multiple regression) |
| 4 | 10.1111/jon.13078 | Amaia Pérez del Barrio | 2023 | Spain; Chile | case-control | Single center | spontaneous intracerebral hemorrhage (sICH) | not mentioned | mortality rate | 17 | CT | 489 | 978 | internal validation | not mentioned | Custom hybrid model (3D Convolutional Neural Network (CNN)+Feedforward Network); I-model (image only CNN model); D-model (feedforward network model with table data only) | 3D brain CT image (1 image type)+clinical variables (41 items, including categorical and numerical variables such as gender, age, smoking, alcohol consumption, hypertension, etc.) |
| 5 | 10.3389/fneur.2024.1406271 | Hu X | 2024 | China | Cohort study | Multicenter | spontaneous intracerebral hemorrhage (sICH) | Conservative treatment or surgery | Poor prognosis (mRS 4-6) | 180 | CT | 276 | 543 | Internal verification+external verification | LASSO regression algorithm | k-nearest neighbor classification (KNN); logistic regression (LR); support vector machine (SVM) | radiomic features（top 10 radiomic features of PRH, PRS, PSH, PSE）; radiological features（black hole, island, blend, and swirl signs） |
| 6 | 10.3389/fsurg.2022.886856 | Daiquan Gao | 2022 | China | case-control | Multicenter | spontaneous intracerebral hemorrhage (sICH) | not mentioned | Neurological deterioration (NIHSS score increase ≥ 4 points or GCS score decrease ≥ 2 points) | 7 | Non-image data | 232 | 535 | internal validation | Single factor analysis (baseline feature difference analysis); Multivariate combination (multiple logistic regression screening for independent risk factors, combined with literature inclusion of variables) | Random Forest Model (unspecified version) | Clinical features+laboratory indicators+imaging indicators (a total of 8: serum calcium, time from onset to emergency department, serum sodium, baseline hematoma volume, 24-hour systolic blood pressure change, age, intraventricular hemorrhage expansion, gender) |
| 7 | 10.1016/j.ejrad.2023.111081 | Yihao Chen | 2023 | China | Cohort study | Multicenter | spontaneous intracerebral hemorrhage (sICH) | Conservative treatment or surgery | Neurological dysfunction (GOS 1-3) | not mentioned | CT |  | 4762 | internal validation | Single factor analysis+multiple factor logistics regression analysis | Convolutional neural networks (CNN)（DenseNet 3-D variant with a depth of 121；gated multimodal unit (GMU)） | Clinical features (22 items: age, sex, GCS score, headache/emesis/coma symptoms, ICH extension to the ventricles, a history of hemorrhagic stroke/ischemic stroke/hypertension/diabetes mellitus/hyperlipidemia/coronary heart disease/heart failure/arrhythmia/anti-coagulant therapy/anti-platelet therapy/smoking/alcohol intake, systolic blood pressure, diastolic blood pressure, and treatment）； Imaging features (CT image features) |
| 8 | 10.1186/s12880-024-01352-y | Hao Zhang | 2024 | China | case-control | Multicenter | spontaneous intracerebral hemorrhage (sICH) | not mentioned | Poor prognosis (mRS 3-6) | 180 | CT | 342 | 666 | external validation | Univariate logistic regression (P<0.05 pre screening); ElasticNet algorithm; MRMR algorithm; Pearson or Spearman methods eliminate redundant features; ICC screening for stable features (ICC ≥ 0.75) | Random Forest (RF) model; EfficientNetV2-L deep learning model | Clinical characteristics (age, sex, hypertension, diabetes, etc.); Radiomics features (1762 items, retained after screening); Imaging features (8 items, such as mixed sign, island sign, etc.); Deep learning features (1000 items, filtered and retained) |
| 9 | 10.1371/journal.pdig.0000493 | Yaobin Ling | 2024 | USA | Cohort study | Multicenter | spontaneous intracerebral hemorrhage (sICH) | Conservative treatment is the main approach | Poor prognosis (mRS 3-6) | 90 | 0 |  | 1741 | internal validation | Recursive partitioning (causal tree/forest, uplift tree/forest); L1 regularized sparse linear model (RuleFit idea); Chi square test for screening significant rules | Uplift Forest (Ensemble Recursive Partition Algorithm); Conditional Generative Adversarial Network (CTGAN); Variational Autoencoder (TVAE); Elastic Net (propensity score matching) | Clinical characteristics (age, race, sex, location of cerebral hemorrhage, blood pressure parameters (SBP, DBP, MAP, PP), laboratory indicators (WBC, hemoglobin, platelet count, etc.), and medical history (hypertension, diabetes, etc.), a total of more than 20 variables |
| 10 | 10.1007/s13760-012-0093-2 | Stevo Lukic | 2012 | Serbia | Cohort study | Single center | spontaneous intracerebral hemorrhage (sICH) | Conservative treatment is the main approach | mortality rate | not mentioned | not mentioned | 594 | 946 | internal validation | Univariate (including variables with a P-value ≤ 0.20, using a backward selection procedure) | Logistic Regression（LR）； Artificial Neural Networks (ANN, standard feedforward and backpropagation neural network, including input layer, two hidden layers, and output layer) | Clinical features (age, gender, pulse blood pressure, mean arterial pressure, three parameters of GCS score (eye opening, speech, motor response), level of consciousness), a total of 8 variables |
| 11 | 10.3389/fneur.2019.00910 | Hsueh-Lin Wang | 2019 | China Taiwan | case-control | Single center | spontaneous intracerebral hemorrhage (sICH) | not mentioned | Poor prognosis (mRS 3-6) | 30 | CT | 289 | 550 | internal validation | InfoGain module (information gain; gain ratio) | Auto WEKA 2.0 (supporting 39 machine learning methods, ultimately selecting the random forest algorithm) | Demographic attributes (age, gender, hypertension history, diabetes history, blood pressure, consciousness level); Radiographic attributes (hematoma volume, location, intraventricular hemorrhage, intraventricular compression, midline structural displacement); Laboratory attributes (blood glucose, AST, ALT, BUN, Cr, BUN/Cr ratio HbAlc、CBC、TG、 Total cholesterol CRP、UA、PT、APTT、hsCRP）， 26 items in total (1 month); 22 items (6 months, excluding white blood cell count compared to 1 month) |
| 12 | 10.1145/3584371.3613002 | Qizhang Feng | 2023 | USA | case-control | MIMIC | spontaneous intracerebral hemorrhage (sICH) | not mentioned | mortality rate | 7 | not mentioned | 921 | 1842 | internal validation | Not mentioned (features selected based on literature and data availability) | Attention based Transformer Model; Logistic Regression; LSTM based Fusion Model | Time series data (vital signs, 7-channel 24-hour data)+aggregated data (196 numerical features, including minimum, maximum, median, and mean values of demographic, comorbidities, laboratory, and chart values) |
| 13 | 10.3389/fneur.2017.00064 | Phan TG | 2017 | Australia | Cohort study | Multicenter | spontaneous intracerebral hemorrhage (sICH) | not mentioned | Poor prognosis (mRS 3-6) | 90 | CT |  | 957 | internal validation | Decision tree recursive partitioning (automatically filtered by the rpart algorithm) | Binary decision tree (rpart); Three point decision tree (rpartScore) | Clinical features (age, gender, systolic blood pressure, blood glucose, GCS, NIHSS); Image features (ICH volume), a total of 6 types of variables |
| 14 | [10.1016/j.jns.2023.120807](https://doi.org/10.1016/j.jns.2023.120807) | Hung L-C | 2023 | China Taiwan | Cohort study | Single center | spontaneous intracerebral hemorrhage (sICH) | Conservative treatment is the main approach | Poor prognosis (mRS 3-6) | during hospitalization | CT | 1333 | 2189 | internal validation | Feature selection (denoising of high-dimensional feature space after BOW text representation, specific method not specified) | XGBoost (ML method); ClinicalBERT+Feedforward Neural Network (DL Method) | Clinical data (age, GCS score, NIHSS score, blood glucose level, ICH location, IVH, ICH volume, etc., a total of 5 variables included in the baseline risk score); Text features (natural language processing features extracted from the current medical history section of admission records, generated through BOW+XGB or BERT modeling) |
| 15 | 10.1007/s00062-021-01040-2 | Chongfeng Duan | 2021 | China | case-control | Single center | spontaneous intracerebral hemorrhage (sICH) | not mentioned | Hematoma enlargement (volume exceeding baseline by 33% or 12.5mL) | 1 | CT | 378 | 756 | internal validation | LASSO regression | Support vector machine（SVM）； Decision tree（DT）； Conditional inference trees（CIT）； Random forest（RF）； k-nearest neighbors（KNN）； Back-propagation neural network（BPNet）； Bayes | Radiomics (texture parameters, 8 in total: 45-7) Correlation、90-7SumVariance、15Percentile-1、Skewness-1、15Percentile-2、5PercentileArea、Texture Strength、Mean Breadth） |
| 16 | 10.1186/s12911-025-02865-4 | Yidan Chen | 2025 | China | case-control | MIMIC | spontaneous intracerebral hemorrhage (sICH) | not mentioned | mortality rate | not mentioned | Non-image data | 572 | 1403 | Internal verification+external verification | LASSO regression; Multivariate logistic regression analysis; Spearman correlation analysis (excluding multicollinearity) | XGBoost (version not mentioned) | Clinical features (7 items in total): GCS motor score, age, GCS eyes score, low-density lipoprotein (LDL), albumin, atrial fibrillation, gender |
| 17 | 10.1177/23969873241260154 | Yutong Chen | 2025 | USA; Germany | Cohort study | Multicenter | spontaneous intracerebral hemorrhage (sICH) | not mentioned | Poor prognosis (mRS 3-6) | 180-730 | CT | 406 | 2682 | Internal verification+external verification | not mentioned | FICHnet (a deep learning based survival analysis model, including architectures such as ResNet34 and DenseNet121) | Image features (NCCT scan images) |
| 18 | 10.1186/s12911-025-02984-y | Xiao-Han Vivian Yap | 2025 | China Taiwan | case-control | Single center | spontaneous intracerebral hemorrhage (sICH) | not mentioned | mortality rate | not mentioned | Non-image data | 570 | 2902 | internal validation | Spearman correlation coefficient; SHAP analysis | Logistic Regression； Random Forest； LightGBM； XGBoost； Multi-layer Perceptron（MLP）， Version not mentioned | Clinical features (36, including age, gender, vital signs, GCS score, pupillary reflex, muscle strength, etc.) FiO2、 Complications, medication use, etc.); Ultimately, 18 key features were adopted |
| 19 | 10.1186/s12967-024-04896-3 | Zhi Geng | 2024 | China | case-control | Multicenter | spontaneous intracerebral hemorrhage (sICH) | not mentioned | Poor prognosis (mRS 3-6) | 90 | CT | 206 | 487 | Internal verification+external verification | Recursive Feature Elimination（RFE） | Support Vector Machine（SVM）； Logistic Regression（LR）； Random Forest（RF）； XGBoost； LightGBM | Clinical features (NIHSS score AST、 Age, white blood cells, hematoma volume, urea nitrogen, neutrophils, glucose, creatinine, systolic blood pressure ALT、 Lymphocytes, diastolic blood pressure, uric acid, GCS score, a total of 15 items |
| 20 | 10.1007/s10072-023-06824-7 | Daiquan Gao | 2023 | China | Cohort study | Multicenter | spontaneous intracerebral hemorrhage (sICH) | Conservative treatment or surgery | Poor prognosis (mRS 3-6) | 90 | CT |  | 412 | internal validation | LASSO regression (multiple factors) | Random Forest Algorithm | Clinical features (12 items): deterioration of neurological function, 24-hour GCS score, baseline GCS score, time from onset to emergency room, blood glucose, 24-hour diastolic blood pressure change, 24-hour hematoma volume change, systemic immune inflammatory index (SII), 24-hour systolic blood pressure change, serum creatinine, serum sodium, age |
| 21 | [10.1016/j.inat.2022.101560](https://doi.org/10.1016/j.inat.2022.101560) | Shinya Sonobe | 2022 | Japan; India | Cohort study | Single center | spontaneous intracerebral hemorrhage (sICH) | Conservative treatment or surgery | Poor prognosis (mRS 3-6) | 28–571 | not mentioned | 100 | 300 | internal validation | not mentioned | Balanced Random Forest algorithm | Patient background (age, sex, BMI, smoking history, alcohol intake, pre-onset disability); Imaging-related finding (affected side, IVH, putaminal hematoma volume, thalamic hematoma volume, EVD, HE); Systemic condition (CTR, sBP, dBP, HR, BT, oxygenation); Neurological finding (consciousness, upper limb paralysis, lower limb paralysis, motor aphasia, sensory aphasia, dysarthria, dysphagia, agnosia, attention deficits, depression, disinhibition); Blood test (Hb, WBC, D-dimer, CRP, HbA1c, LDL, TG, Alb, CK, LDH, GGT, BUN, Cr, UA) (5 categories, 43 items) |
| 22 | 10.1177/25166085221127861 | Girish Menon | 2022 | India | Cohort study | Single center | spontaneous intracerebral hemorrhage (sICH) | Conservative treatment or surgery | Poor prognosis (mRS 3-6) | not mentioned | CT |  | 2000 | internal validation | XGBoost Feature Importance Score | XGBoost (implemented on scikit learn platform) | Clinical features (age, gender, comorbidities, etc., a total of 19 items) |
| 23 | [10.1016/j.ibmed.2025.100237](https://doi.org/10.1016/j.ibmed.2025.100237) | Ming Jie | 2025 | Singapore | Cohort study | Single center | spontaneous intracerebral hemorrhage (sICH) | not mentioned | Deterioration of neurological function (GCS decrease ≥ 2 points); Death during hospitalization | 2 | CT | 257 | 638 | internal validation | Recursive Feature Elimination Cross Validation (RFECV); Single factor statistical analysis+Extra Trees classifier based on Gini impurity ranking (combination of filtering and embedding methods) | Random Forest; Extra Trees； CatBoost； Blended Soft Voting Integrated Model (PyCaret 3.0, based on scikit learn, XGBoost, CatBoost frameworks) | Clinical features (age, initial GCS, GCS severity, hypertension, ICH score, PT, APTT, POCT blood glucose HCT、WBC）； Radiological features (hematoma volume, intraventricular hemorrhage (IVH), midline shift); 12 items in total |
| 24 | 10.1016/j.nicl.2023.103378 | Xiaona Xia | 2023 | China | Cohort study | Multicenter | spontaneous intracerebral hemorrhage (sICH) | Conservative treatment or surgery | Poor prognosis (mRS 3-6) | 90 | CT | 308 | 936 | external validation | Univariate analysis (p<0.05); Multi factor logistic regression | Deep convolutional neural networks (deep learning convolutional neural networks) | Clinical features (baseline GCS score, age ≥ 80 years, ICH volume ≥ 30mL, initial IVH, HE); Radiomics features (dHU) |
| 25 | 10.1016/j.acra.2024.07.025 | Dan Wang | 2025 | China | Cohort study | Multicenter | spontaneous intracerebral hemorrhage (sICH) | not mentioned | Poor prognosis (mRS 4-6) | 90 | CT | 1194 | 3294 | internal validation | Single factor logistic regression; Multivariate logistic regression; LASSO regression; One way analysis of variance (ANOVA) | ResNet50 (deep learning model); logistic regression | Clinical features (age, IVH, hematoma volume, PHE volume, admission GCS score, etc.); Deep Learning Features (15) |
| 26 | 10.3389/fneur.2021.790682 | Wei Chen | 2022 | China; USA | Cohort study | Single center | spontaneous intracerebral hemorrhage (sICH) | Conservative treatment or surgery | mortality rate | not mentioned | CT | 645 | 3240 | internal validation | Five fold cross validation screening of 13 most influential laboratory parameters | Logistic regression; Classification and regression trees; Random forest; eXtreme Gradient Boosting（XGBoost）， The XGBoost model was ultimately chosen | Clinical features (5 ICH scoring variables: GCS score, ICH volume IVH、 Bleeding site, age); Laboratory parameters (13 items: blood glucose, creatinine, white blood cell count, low-density lipoprotein, prothrombin time, aspartate aminotransferase, lymphocyte percentage, chloride, potassium, red blood cell distribution width, uric acid, phosphorus, alanine aminotransferase to aspartate aminotransferase ratio) |
| 27 | [10.1016/j.compbiomed.2023.107397](https://doi.org/10.1016/j.compbiomed.2023.107397) | Yantao Xing | 2023 | China | case-control | Single center | spontaneous intracerebral hemorrhage (sICH) | not mentioned | Neurological dysfunction (GOS 1-3) | not mentioned | not mentioned | 109 | 232 | internal validation | SHAP value (used to quantify feature importance) | XGBoost； Comparison model: K-Nearest Neighbor（KNN）、Support Vector Machine（SVM）、Random Forests（RF） | SKNA signal features (time domain, frequency domain, nonlinear features, a total of 39); HRV features (as a comparative method, not used for modeling) |
| 28 | 10.1016/j.compmedimag.2024.102430 | C. Yalcin | 2024 | Spain | case-control | Single center | spontaneous intracerebral hemorrhage (sICH) | not mentioned | Hematoma enlargement | 1 | CT | 35 | 70 | internal validation | not mentioned | EfficientNet B0； Comparison models: ResNet34, DenseNet121 | Imaging features (initial non enhanced CT scan and corresponding lesion annotation) |
| 29 | 10.1007/s12028-020-00982-8 | Hall AN | 2021 | USA | Cohort study | Multicenter | spontaneous intracerebral hemorrhage (sICH) | not mentioned | Poor prognosis (mRS 4-6) | 14 | CT | 816 | 1112 | Internal verification+external verification | Automatic selection using decision tree algorithm (based on Gini Impurity index); Random Forest Algorithm Based on Variable Importance | Decision tree (R package "rpart"); Random Forest (R package "randomForest"), R version 3.5.3 | Clinical characteristics: Glasgow Coma Scale (GCS) score, hematoma volume, hematoma enlargement, intraventricular hemorrhage, ICH score, age, pre disease mRS score, gender, race, diabetes history, hypertension history, aspirin use, warfarin use, etc., a total of 19 characteristics (Institution 1); The last 15 characteristics after harmonization (Institution 2) |
| 30 | 10.1007/s12975-021-00891-8 | Jawed Nawabi | 2021 | Germany; Switzerland; Italy | Cohort study | Multicenter | spontaneous intracerebral hemorrhage (sICH) | not mentioned | Poor prognosis (mRS 3-6) | 1 | CT | 3371 | 6240 | internal validation | Feature importance analysis based on Gini impurity metric (separate feature selection in each outer loop sample segmentation of 5-fold cross validation) | Random Forest Algorithm (Python scikit learn environment v0.20.3) | Radiomics features (1218, including 252 first-order features, 902 texture features, and 14 shape features); Clinical parameters (including only decompressive craniectomy) |
| 31 | 10.3390/brainsci14060618 | Manli Xu | 2024 | China | case-control | Single center | spontaneous intracerebral hemorrhage (sICH) | Conservative treatment or surgery | Neurological dysfunction (GOS 1-3) | not mentioned | CT | 145 | 294 | internal validation | not mentioned | ICH Net (including ResNet50 visual encoder, BioClinical BERT text encoder, Cross Modal Attention Fusion (CMAF) module, Multi Head Self Attention Fusion (MHSAF) module) | Clinical text data (age, gender, GCS score, etc.)+CT imaging features (bleeding location, volume, etc.), a total of two types of variables |
| 32 | 10.3390/diagnostics14242827 | Fiona Dierksen | 2024 | USA; Germany | Cohort study | Multicenter | spontaneous intracerebral hemorrhage (sICH) | Conservative treatment is the main approach | Poor prognosis (mRS 4-6) | 90 | CT |  | 6760 | external validation | MRMR； pMIM； RIDGE； HClust； PCA； No Feature Selection (noFS) | Elastic Net-regularized logistic regression（ElNet）； Random Forest（RF）； Support Vector Machine with sigmoid kernel（SVM sig）； Support Vector Machine with radial kernel（SVM rad）； Naïve Bayes（NBayes）； XGBoost（XGB）； All based on pyradiomics 2.2.0 | Radiomics (ICH Radiomics 1130 Characteristics; 2260 features of ICH+PHE radiomics; Clinical variables (clinical predictive factors at admission, such as GCS, NIHSS, etc.); Radiomics+Clinical Variables |
| 33 | 10.1016/j.jstrokecerebrovasdis.2021.106234 | Mervyn Jun Rui Lim | 2022 | Singapore | Cohort study | Single center | spontaneous intracerebral hemorrhage (sICH) | not mentioned | Poor prognosis (mRS 3-6) | 90 | CT | 418 | 2316 | internal validation | Filter based method (statistical combination); Wrap based methods (Recursive Feature Elimination (RFE), Boruta); Embedded methods (Lasso, Random Forest, Light Gradient Boosting Machine (LGBM), Extreme Gradient Boosting (XGBoost)); Dimensionality reduction methods (Principal Component Analysis (PCA), Autoencoder, t-Distributed Stochastic Neighborhood Embedding (tSNE), Uniform Manifold Approximation and Projection (UMAP)) | Support Vector Machine (SVM); Deep Neural Network (DNN) | Continuous variables: age, hematoma volume, midline shift, hemoglobin, heart rate, mean arterial pressure, systolic blood pressure, blood glucose, total white blood cell count, platelet count, prothrombin time, international standardized ratio, creatinine, sodium, potassium, etc; <br>Classified variables: gender, race, activity of daily living, community activity status, history of cognitive impairment, past history of cerebral hemorrhage, hypertension, diabetes, hyperlipidemia, smoking status, medication (anticoagulation, antiplatelet), dialysis dependence, hematoma location (supratentorial/infratentorial, cortical/subcortical/brainstem/cerebellum), CT spot sign, ventricular expansion, Glasgow Coma Scale (GCS), etc |
| 34 | 10.3390/jpm12010112 | Rui Guo | 2022 | China | Cohort study | Single center | spontaneous intracerebral hemorrhage (sICH) | Conservative treatment or surgery | Poor prognosis (mRS 3-6) | 90 | CT | 1644 | 9012 | internal validation | Single factor analysis; Recursive Feature Elimination Cross Validation (RFECV) | Logistic regression (LR); Logistic regression cross validation (LRCV); Support Vector Machine (SVM); Random Forest (RF); Extreme Gradient Boosting (XGBoost); Category Enhancement (CatBoost) | Clinical features (age, gender, GCS, blood pressure, etc., a total of 28 items); Imaging features (hematoma location, volume, IVH, etc., a total of 5 items); Laboratory characteristics (blood glucose, creatinine, D-dimer, etc., a total of 18 items), with a total of 51 variables |
| 35 | 10.1016/j.ejrad.2024.111543 | Lu Shi | 2024 | China | case-control | Single center | spontaneous intracerebral hemorrhage (sICH) | not mentioned | mortality rate | 7 days | CT | 411 | 792 | internal validation | Single factor logistic regression; LASSO | Random Forest | Clinical features (14 items); Radiomics features (67 items, including 11 semantic features and 56 shape features); Clinical features+radiomics features (81 items) |
| 36 | 10.1002/cpe.70042 | Qingqing Wu | 2025 | China | case-control | Single center | spontaneous intracerebral hemorrhage (sICH) | conservative treatment | Poor prognosis (mRS 3-6) | 90 | CT |  | 780 | internal validation | Spearman Correlation Analysis; Principal Component Analysis | Random Forest (RF)； XGBoost； logistic regression (LR)； LGBoost； AdaBoost | Clinical characteristics (age, gender, history of hypertension, stroke, history of diabetes, history of coronary heart disease, interval between onset and first imaging examination, about 20 items in total); Imaging features (hematoma volume, edema volume, shape features, CT first-order statistical features, etc., totaling about 40 items), totaling 60 items |
| 37 | 10.1016/j.jstrokecerebrovasdis.2022.106475 | Xin Qi | 2022 | China | case-control | Single center | spontaneous intracerebral hemorrhage (sICH) | not mentioned | Poor prognosis (mRS 3-6) | 90 | CT | 120 | 236 | internal validation | SelectPercentile； SelectFromModel； Iterative screening method | Logistic Regression Algorithm (LR) | Radiomics features (2260 radiomics features from hematoma and hematoma+surrounding tissue, ultimately retaining 8 features each) |
| 38 | [10.1016/j.crad.2023.10.002](https://doi.org/10.1016/j.crad.2023.10.002) | Q. Chen | 2024 | China | case-control | Multicenter | spontaneous intracerebral hemorrhage (sICH) | not mentioned | Hematoma enlargement | not mentioned | CT |  | 2549 | internal validation | MRMR algorithm; LASSO algorithm | regularised L1 logistic regression（L1 logit）； decision tree（DT）； support vector machine（SVM）； AdaBoost | Radiomics features (2D and 3D radiomics features); Clinical risk factors (clinical risk factors closely related to HE, not specifically listed) |
| 39 | 10.3389/fneur.2020.610531 | Ximing Nie | 2021 | China Hong Kong | case-control | MIMIC | spontaneous intracerebral hemorrhage (sICH) | not mentioned | mortality rate | not mentioned | Non-image data | 2681 | 5320 | internal validation | Clinical experience manual screening combined with random forest algorithm (selecting variables with feature scores>0.0005 in descending order of importance) | nearest neighbors; decision tree; neural net; AdaBoost; random forest; gcForest | Clinical features (age, gender, GCS score, APACHE II score, vital signs, etc., totaling 72 items) |
| 40 | 10.3389/fneur.2021.655800 | Yang W-S | 2021 | China | Cohort study | Single center | spontaneous intracerebral hemorrhage (sICH) | not mentioned | Poor prognosis (mRS 3-6) | 90 | CT | 240 | 620 | not mentioned | Univariate analysis (P ≤ 0.1)+multivariate logistic regression | Logistic regression model | Clinical features (age, baseline GCS score, presence of IVH on initial CT, baseline ICH volume, subarachnoid hemorrhage); Dynamic features (hematoma enlargement, IVH growth); NCCT imaging markers (mixed sign, black hole sign, island sign), a total of 9 items |
| 41 | 10.3390/jcm12041580 | Kangwei Zhang | 2023 | China | Cohort study | Multicenter | spontaneous intracerebral hemorrhage (sICH) | surgical treatment | Poor prognosis (mRS 4-6) | 90 | CT | 179 | 280 | Internal verification+external verification | 12 feature selection algorithms (CIFE, CMIM, DISR, FastICA, ICAP, JMI, Lasso, MIM, NMF, None, PCA, TruncatedSVD) | 9 machine learning models (Adaboost, DET, EXT, KNN, LR, MLP, RF, SVM, Xgboost) | Clinical features (age, gender, admission GCS, IVH, MLS, deep ICH); Radiomics features (108, 22 after screening); Clinical features+radiomics features |
| 42 | 10.3389/fneur.2024.1494934 | Pan B | 2025 | China | Cohort study | Multicenter | spontaneous intracerebral hemorrhage (sICH) | Conservative treatment or surgery | Poor prognosis (mRS 3-6) | 90 | CT | 207 | 836 | internal validation | Single factor analysis; LASSO regression; Pearson correlation analysis | complementary naive bayes (CNB)； support vector machine (SVM)； gaussian naive bayes (GNB)； multilayer perceptron (MLP)； extreme gradient boosting (XGB) | Demographic characteristics (gender, age); Past medical history (hypertension, use of antithrombotic drugs, diabetes, heart disease, cerebral infarction, cerebral hemorrhage, trauma, uremia, chronic liver disease, smoking, drinking); Baseline vital signs (systolic blood pressure, diastolic blood pressure, GCS score); Baseline disease characteristics (time from onset to emergency, emergency antihypertensive treatment, bleeding site, hematoma volume); Laboratory tests (INR, PT, APTT, TT, Fbg, D-dimer,...) HGB、PLT、 Blood sugar); Treatment related indicators (systolic blood pressure changes, diastolic blood pressure changes, treatment methods), a total of 6 categories and 30 items |
| 43 | 10.1016/j.bspc.2022.103656 | Zhi-Ri Tang | 2022 | China | case-control | Single center | spontaneous intracerebral hemorrhage (sICH) | not mentioned | Hematoma enlargement (volume exceeding baseline by 33% or 6mL) | 3 | CT | 137 | 223 | internal validation | not mentioned | K-nearest neighbors matting； Modified deep residual network (DRN, modified based on ResNet-34) | Imaging features (features of brain CT scan images preprocessed by KNN matting) |
| 44 | 10.1371/journal.pone.0296616 | Misra S | 2024 | India; USA; UK | Cohort study | Single center | spontaneous intracerebral hemorrhage (sICH) | not mentioned | Poor prognosis (mRS 3-6) | 90 | Non-image data | 672 | 1172 | internal validation | Single factor analysis (p<0.1); Multi factor (stepwise regression backwards); Random Forest Algorithm (Shapley Value Evaluation of Variable Importance) | Logistic regression; Cox regression; Random Forest Algorithm (scikit learn package, 1000 estimators) | Clinical variables (age, gender, surgical condition, hypertension, diabetes, NIHSS score, GCS score, ICH volume, intraventricular hemorrhage, systolic blood pressure, diastolic blood pressure, etc.); Protein biomarkers (22 types, such as UCH-L1, alpha-2-macroglobulin, SerpinA11, MMP-2, IGFBP-3, MMP-9, etc.) |
| 45 | 10.1016/j.wneu.2017.02.082 | Gupta VP | 2017 | USA | Cohort study | Single center | spontaneous intracerebral hemorrhage (sICH) | not mentioned | Poor prognosis (mRS 4-6) | 90 | 0 | 436 | 880 | external validation | Random Forest machine learning technology (based on normalized "importance" score ≥ 0.01) | Random ForestClassifier（Python 2.7；SciPy stack 0.18.0；sklearn.ensemble）； Linear regression (Python 2.7; SciPy stack 0.18.0; sklearn. linear_model) | Clinical features (GCS, NIHSS, APACHE II Physiologic score, pre disease mRS, hematoma volume (3-month model only)), a total of 5 items (3 months)/4 items (12 months) |
| 46 | 10.1016/j.wneu.2023.11.095 | Mervyn Jun Rui Lim | 2024 | Singapore | Cohort study | Single center | spontaneous intracerebral hemorrhage (sICH) | surgical treatment | Poor prognosis (mRS 3-6) | 90 | CT | 247 | 1775 | internal validation | Random Forest Feature Selection | Deep neural network; Random forest; Support Vector Machine (SVM); XGBoost； logistic regression | Clinical features (Glasgow Coma Scale score, age, hematoma volume, hematoma location, ventricular expansion, hypertension status, independence of daily living activities, midline shift, total white blood cell count, glucose, sodium, hemoglobin, platelet count, etc.) |
| 47 | 10.1111/j.1468-1331.2010.02955.x | S.-Y. Peng | 2010 | China Taiwan | case-control | Single center | spontaneous intracerebral hemorrhage (sICH) | not mentioned | mortality rate | 30 | CT | 620 | 4230 | internal validation | Logistic regression uses stepwise forward/backward selection method (P<0.05 included, P>0.1 excluded); ANN and SVM use Consistent Subset Evaluation (CSE) combined with exhaustive search method | Random Forest (300 decision trees, randomly selecting 5 variable splitting nodes); Artificial Neural Network (multi-layer perceptron, backpropagation algorithm); Support Vector Machine； Logistic Regression； ICH score | Clinical factors (age, medical history (hypertension, diabetes, anemia, dialysis dependence, previous stroke, ischemic heart disease), level of consciousness (GCS score), blood pressure); Radiological variables (hematoma volume, bleeding site, intraventricular hemorrhage, hydrocephalus, pineal gland displacement); Laboratory data (hemoglobin, blood glucose), totaling approximately 15 variables |
| 48 | 10.1093/qjmed/hcl107 | O. Takahashi | 2006 | Japan; USA | Cohort study | Single center | spontaneous intracerebral hemorrhage (sICH) | Conservative treatment is the main approach | mortality rate | not mentioned | CT | 70 | 347 | not mentioned | Univariate analysis (p<0.25) combined with clinical significance | Classification and Regression Tree (CART) 5.0； Multiple logistic regression | Clinical features (age, level of consciousness, blood pressure, body temperature, medical history, etc., a total of 29 latent variables); Imaging features (hematoma volume, intraventricular hemorrhage, bleeding site, etc.) |
| 49 | 10.1007/s10143-024-03115-3 | Ajay Hegde | 2024 | India; USA | Cohort study | Single center | spontaneous intracerebral hemorrhage (sICH) | not mentioned | Poor prognosis (mRS 4-6) | 90 | CT | 2108 | 4000 | internal validation | Cram é r's V correlation statistic (used to evaluate the correlation between input variables and target variables) | Google AutoML © Tables (supervised deep learning, based on Adanet Autoensembler ensemble algorithm, including linear, feedforward neural networks, gradient boosting decision trees, and other architectures); Logistic Regression (LR, using R language glm function) | Clinical characteristics+radiological characteristics, 17 in total:<br>Clinical characteristics (11): age, gender, hypertension, diabetes, alcohol intake, smoking, antiplatelet drug use, heart rate, systolic blood pressure, diastolic blood pressure, Glasgow coma score (GCS), admission blood glucose<br>Radiological characteristics (6): hematoma volume (calculated by abc/2 formula), bleeding site (basal ganglia, thalamus, lobe, cerebellum, brain stem, primary ventricular hemorrhage), side (right, left, midline, bilateral), intraventricular expansion, hydrocephalus |
| 50 | 10.2147/IJGM.S408725 | Ruting Bo | 2023 | China | case-control | Single center | spontaneous intracerebral hemorrhage (sICH) | not mentioned | Hematoma enlargement (volume exceeding baseline by 33% or 6mL) | 3 | CT | 24 | 304 | internal validation | K-highest score（K_Best） | Inception_v3（CNN）； SVM | Clinical characteristics (15 items: gender, age, blood pressure, ADL index, history of hypertension/heart disease/diabetes, platelet count, etc.); Radiomics features (107 items); CNN features (6144 items) |
| 51 | 10.1161/JAHA.124.036447 | Matsumoto K. | 2024 | Japan | Cohort study | Single center | spontaneous intracerebral hemorrhage (sICH) | conservative treatment | Neurological dysfunction (GOS 1-3) | 180 | CT | 228 | 1581 | external validation | L1 regularization (LASSO) for variable selection in logistic regression models | ResNet（3D CNN）； L1 regularized logistic regression | Image model: 3D CT image features; Non specialized model: imaging features+non specialized clinical data (demographic, physiological, laboratory); Specialized model: Non specialized data+specialized assessment (GCS, ICH volume, intraventricular hemorrhage, bleeding site) |
| 52 | 10.1007/s10143-022-01802-7 | Trevisi G. | 2022 | Italy | Cohort study | Multicenter | spontaneous intracerebral hemorrhage (sICH) | Conservative treatment is the main approach | mortality rate | not mentioned | Non-image data |  | 324 | internal validation | Boruta algorithm (fully correlated feature selection based on random forest) | Random Forest（Python scikit-learn） | 10 selected features: GCS, Charlson comorbidity index, ICH score, ICH volume, pupil status, brainstem involvement, age, anticoagulant/antiplatelet drugs, intraventricular hemorrhage, cerebellar region |
| 53 | 10.1038/s41598-024-65128-8 | Mao BJ | 2024 | China | Cohort study | Multicenter | spontaneous intracerebral hemorrhage (sICH) | conservative treatment | mortality rate | 14 | CT | 716 | 3082 | Internal verification+external verification | LASSO regression (λ coordinate descent method) | XGBoost； Logistic regression; KNN； AdaBoost； Random Forest | 14: GCS, SOFA, anticoagulant use, mannitol use, vasoactive drugs, mechanical ventilation, body temperature, blood sodium, blood potassium RDW、 Blood chlorine, heart failure, oxygen saturation, surgical intervention |

**Table S2.** Pooled sensitivity and specificity of machine learning for predicting hematoma expansion

| Subgroup analysis | Model | n | Training set | |  | Validation set | |
| --- | --- | --- | --- | --- | --- | --- | --- |
|  |  |  | Sensitivity (95%CI) | Specificity (95%CI) | n | Sensitivity (95%CI) | Specificity (95%CI) |
| Clinical |  |  |  |  |  |  |  |
|  | LR | 6 | 0.72 (0.67-0.76) | 0.65 (0.59-0.72) | 6 | 0.69 (0.60-0.77) | 0.66 (0.59-0.73) |
| Overall |  | 6 | 0.72 (0.67-0.76) | 0.65 (0.59-0.72) | 6 | 0.69 (0.60-0.77) | 0.66 (0.59-0.73) |
| Radiomics |  |  |  |  |  |  |  |
|  | LR | 7 | 0.84 (0.81-0.87) | 0.87 (0.80-0.92) | 9 | 0.74 (0.68-0.80) | 0.83 (0.75-0.88) |
| Overall |  | 19 | 0.82 (0.78-0.85) | 0.82 (0.77-0.86) | 17 | 0.76 (0.71-0.80) | 0.79 (0.74-0.84) |
| Radiomics+Clinical |  |  |  |  |  |  |  |
|  | LR | 10 | 0.81 (0.75-0.85) | 0.78 (0.68-0.85) | 14 | 0.76 (0.70-0.81) | 0.79 (0.73-0.83) |
| Overall |  | 15 | 0.81 (0.77-0.85) | 0.76 (0.70-0.82) | 19 | 0.77 (0.72-0.80) | 0.77 (0.73-0.81) |

**Table S3.** Pooled sensitivity and specificity of machine learning for predicting poor functional outcomes

| Subgroup analysis | Model | n | Training set | | Validation set | | |
| --- | --- | --- | --- | --- | --- | --- | --- |
|  |  |  | Sensitivity (95%CI) | Specificity (95%CI) | n | Sensitivity (95%CI) | Specificity (95%CI) |
| Clinical |  |  |  |  |  |  |  |
|  | LR | 4 | 0.80 (0.72-0.86) | 0.76 (0.73-0.79) | 5 | 0.79 (0.74-0.83) | 0.75 (0.71-0.79) |
| Overall |  | 8 | 0.81 (0.76-0.86) | 0.80 (0.73-0.86) | 13 | 0.74 (0.68-0.80) | 0.84 (0.75-0.90) |
| Radiomics |  |  |  |  |  |  |  |
|  | LR |  |  |  | 5 | 0.74 (0.65-0.81) | 0.85 (0.64-0.94) |
| Overall |  | 6 | 0.79 (0.67-0.87) | 0.81 (0.76-0.85) | 13 | 0.74 (0.68-0.80) | 0.84 (0.75-0.90) |
| Radiomics+Clinical |  |  |  |  |  |  |  |
|  | LR | 6 | 0.79 (0.65-0.89) | 0.83 (0.78-0.87) |  |  |  |
|  | RF | 5 | 0.58 (0.28-0.83) | 0.80 (0.59-0.92) |  |  |  |
| Overall |  | 14 | 0.64 (0.46-0.79) | 0.78 (0.69-0.86) |  |  |  |

**Table S4.** Pooled sensitivity and specificity of machine learning for predicting mortality

| Subgroup analysis | Model | n | Training set | |  | Validation set | |
| --- | --- | --- | --- | --- | --- | --- | --- |
|  |  |  | Sensitivity (95%CI) | Specificity (95%CI) | n | Sensitivity (95%CI) | Specificity (95%CI) |
| Clinical |  |  |  |  |  |  |  |
|  | XGB |  |  |  | 5 | 0.72 (0.60-0.82) | 0.77 (0.56-0.89) |
| Overall |  | 15 | 0.67 (0.59-0.75) | 0.77 (0.71-0.82) | 6 | 0.71 (0.60-0.79) | 0.76 (0.58-0.87) |
| Radiomics |  |  |  |  |  |  |  |
| Overall |  | 6 | 0.87 (0.81-0.92) | 0.87 (0.78-0.92) | 4 | 0.87 (0.67-0.95) | 0.86 (0.70-0.94) |
| Radiomics+Clinical |  |  |  |  |  |  |  |
|  | LR | 4 | 0.81 (0.31-0.97) | 0.91 (0.78-0.97) | 5 | 0.63 (0.20-0.92) | 0.95 (0.81-0.99) |
| Overall |  | 9 | 0.80 (0.63-0.90) | 0.84 (0.75-0.90) | 10 | 0.76 (0.51-0.91) | 0.87 (0.77-0.93) |

**
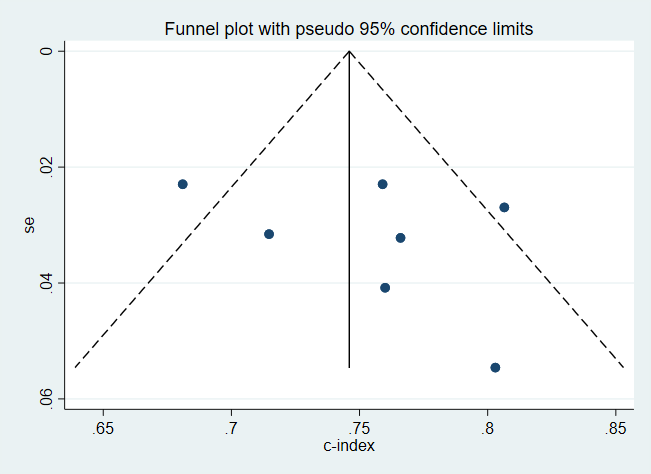
**

**Figure S1** Meta-analysis funnel plot for clinical feature-based models for predicting hematoma expansion in the training set


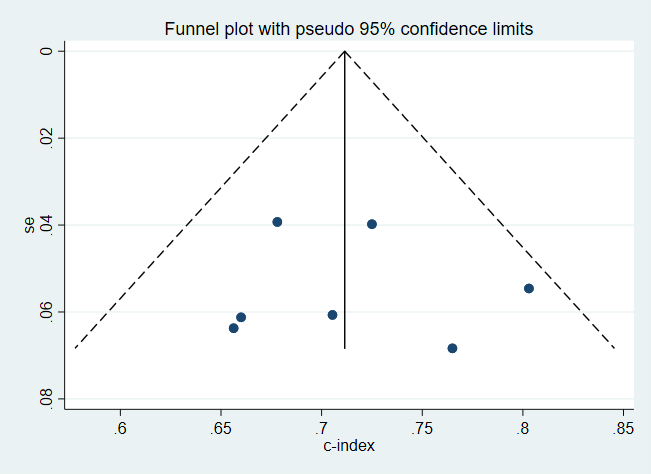


**Figure S2** Meta-analysis funnel plot for clinical feature-based models for predicting hematoma expansion in the validation set


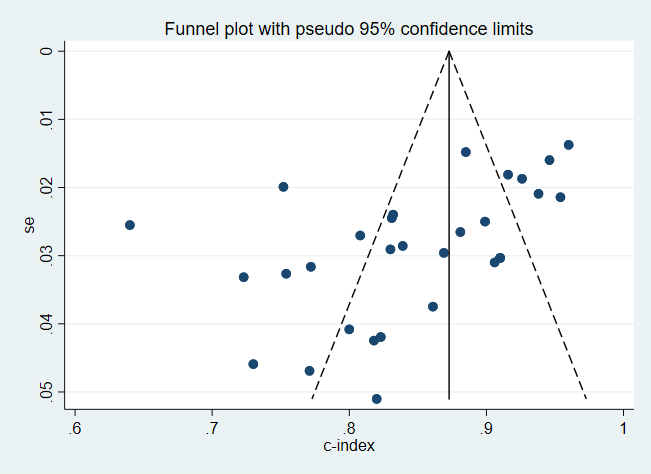


**Figure S3** Meta-analysis funnel plot for radiomics-based models for predicting hematoma expansion in the training set


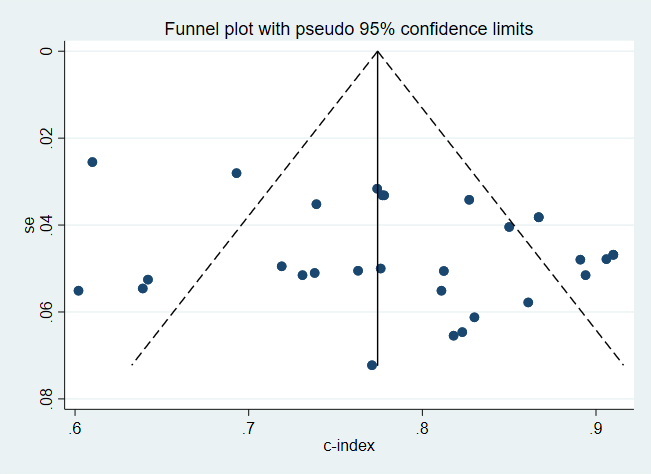


**Figure S4** Meta-analysis funnel plot for radiomics-based models for predicting hematoma expansion in the validation set


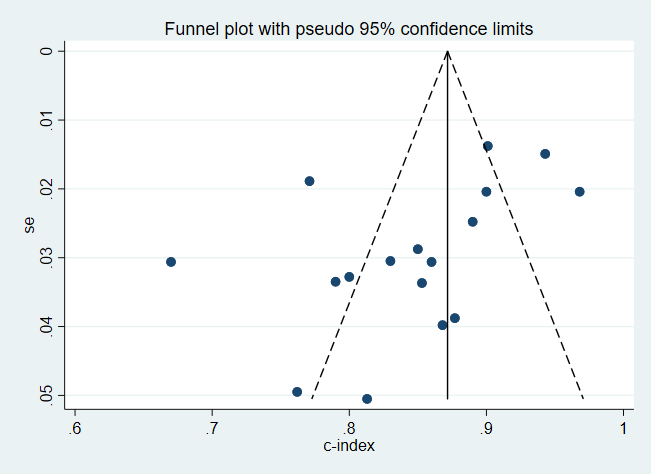


**Figure S5** Meta-analysis funnel plot for combined clinical-radiomics models for predicting hematoma expansion in the training set


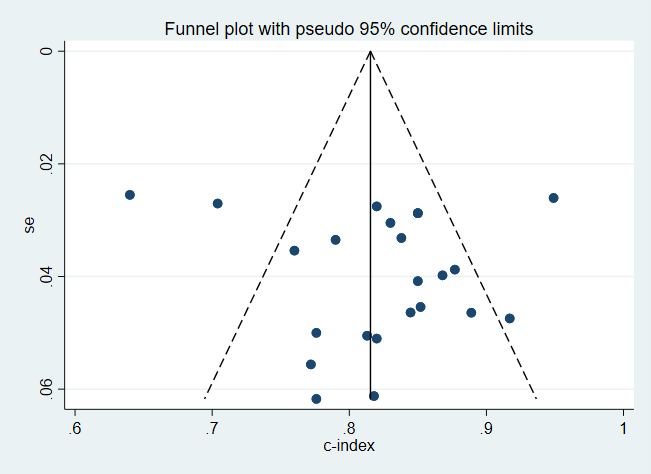


**Figure S6** Meta-analysis funnel plot for combined clinical-radiomics models for predicting hematoma expansion in the validation set


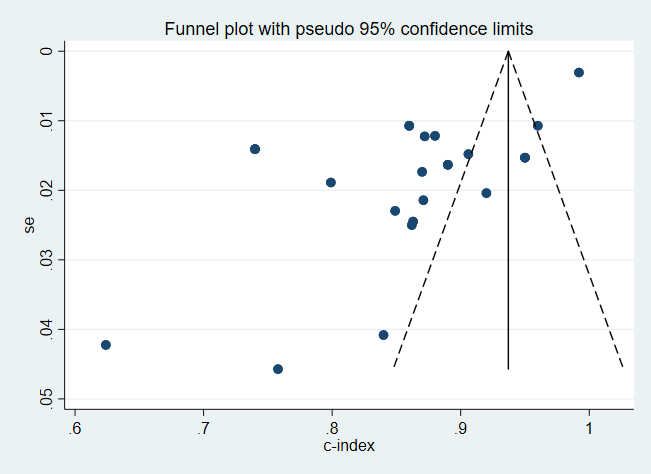


**Figure S7** Meta-analysis funnel plot for clinical feature-based models for predicting poor functional outcome in the training set


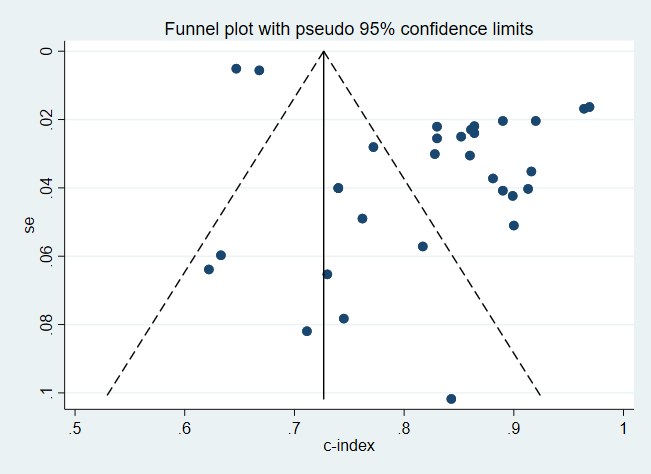


**Figure S8** Meta-analysis funnel plot for clinical feature-based models for predicting poor functional outcome in the validation set


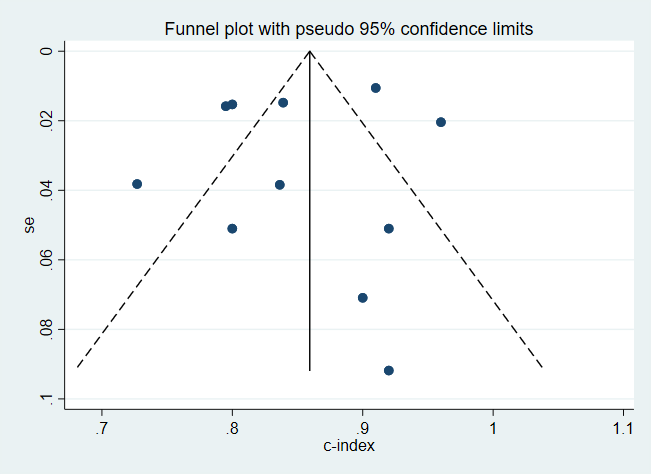


**Figure S9** Meta-analysis funnel plot for radiomics-based models for predicting poor functional outcome in the training set


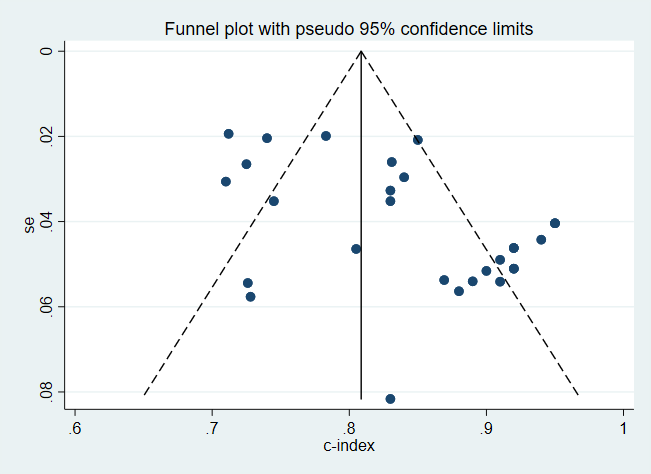


**Figure S10** Meta-analysis funnel plot for radiomics-based models for predicting poor functional outcome in the validation set


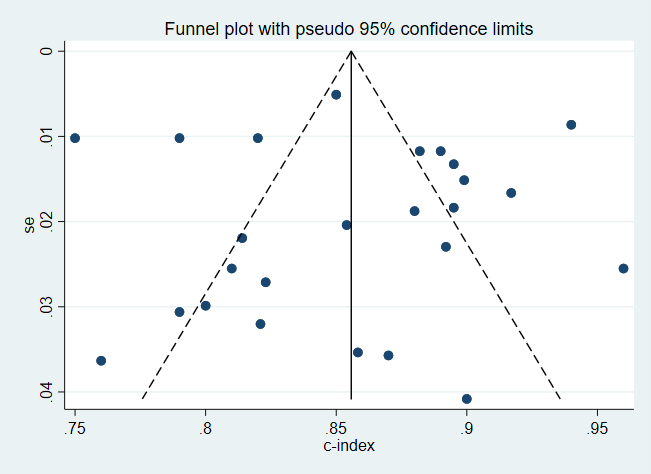


**Figure S11** Meta-analysis funnel plot for combined clinical-radiomics models for predicting poor functional outcome in the training set


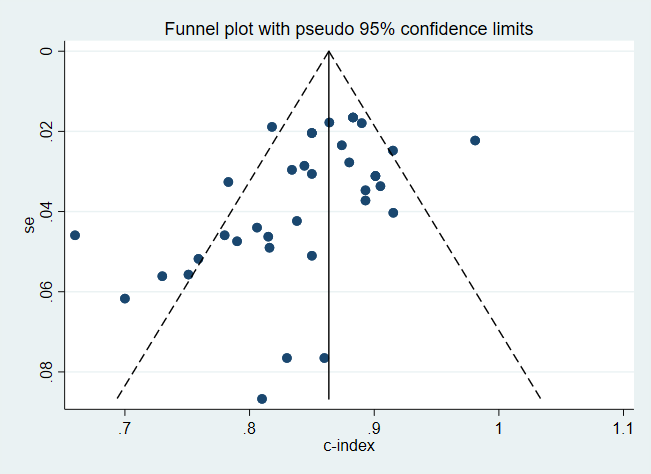


**Figure S12** Meta-analysis funnel plot for combined clinical-radiomics models for predicting poor functional outcome in the validation set


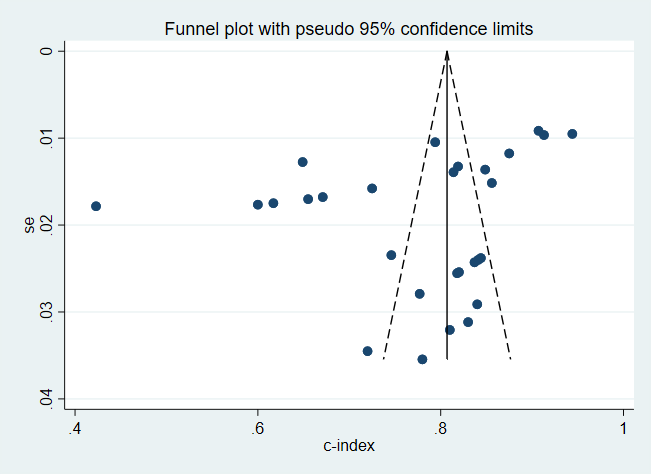


**Figure S13** Meta-analysis funnel plot for clinical feature-based models for predicting mortality in the training set


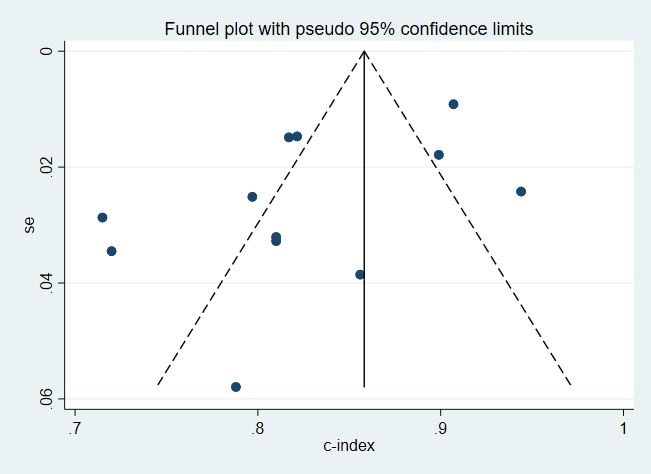


**Figure S14** Meta-analysis funnel plot for clinical feature-based models for predicting mortality in the validation set


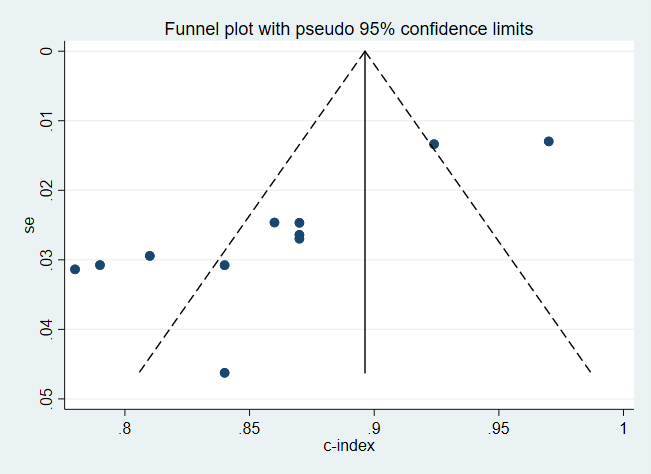


**Figure S15** Meta-analysis funnel plot for combined clinical-radiomics models for predicting mortality in the training set


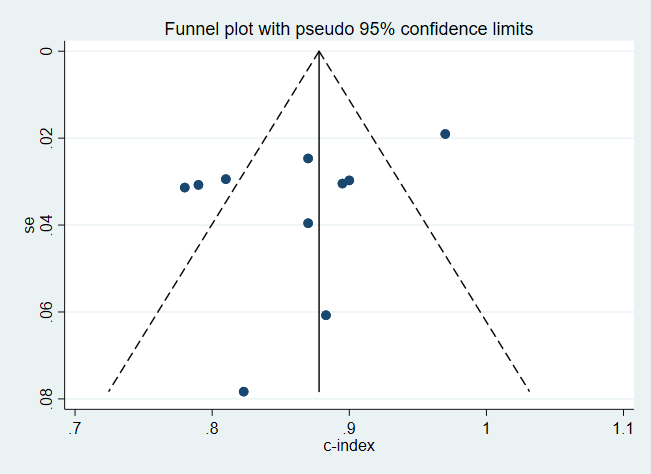


**Figure S16** Meta-analysis funnel plot for combined clinical-radiomics models for predicting mortality in the validation set
